# Enzymatic post-translational modifications of proteins in chronic kidney disease: mechanisms, regulation, and clinical significance

**DOI:** 10.3389/fphar.2025.1678812

**Published:** 2025-10-08

**Authors:** Minlong Wei, Jinyun Lin, Yi Zeng, Xiaojuan Wang, Jialu Wen, Jing Wang, Wei Zou, Kang Tu, Menghua Liu, Juan Li

**Affiliations:** ^1^ Key Laboratory of Drug Metabolism Research and Evaluation of the State Drug Administration, Guangdong Provincial Key Laboratory of New Drug Screening, School of Pharmaceutical Sciences, Southern Medical University, Guangzhou, China; ^2^ Southern Medical University Hospital of Integrated Traditional Chinese and Western Medicine, Southern Medicine University, Guangzhou, China; ^3^ School of Traditional Chinese Medicine, Southern Medical University, Guangzhou, China; ^4^ Changsha Research and Development Center on Obstetric and Gynecologic Traditional Chinese Medicine Preparation, Hunan Provincial Maternal and Child Healthcare Hospital, Changsha, Hunan, China; ^5^ School of Nursing, Southern Medical University, Guangzhou, Guangdong, China

**Keywords:** enzymatic post-translational modifications, chronic kidney disease, therapeutictargets, methylation, acetylation, lactylation

## Abstract

Chronic kidney disease (CKD) involves intricate pathological mechanisms that currently lack definitive therapeutic interventions to halt disease progression. Increasing evidence suggests that enzymatic post-translational modifications (ePTMs) of proteins play an important role in CKD. As a dynamic and reversible type of PTM, ePTMs offer advantages such as enzyme-specific catalysis, high reversibility, and precise regulation. Various forms of ePTMs have been reported in CKD, including methylation, acetylation, ubiquitination, enzymatic glycosylation, lactylation, palmitoylation, crotonylation, SUMOylation, and prenylation. Given the critical roles of these ePTMs in CKD, this review summarizes their molecular mechanisms in disease progression, explores their potential as diagnostic markers and therapeutic targets, and highlights advances in small-molecule drugs targeting ePTMs. It is important to note that most ePTMs remain in the early stages of research, with evidence of cross-regulation and synergistic effects among different modifications. Further investigation will require more basic studies and clinical trials. This review aims to help bridge the gap between basic research and clinical application of ePTMs in CKD, and to support the development of more effective treatment strategies.

## 1 Introduction

Chronic kidney disease (CKD), characterized by progressive loss of renal function, has become a global public health problem that affects approximately 10% of the world’s population (Miguel et al., 2025; Romagnani et al., 2025). With the disease progressing, patients with CKD usually face the risk of multiple adverse outcomes, including cognitive impairment, cardiovascular events, end-stage kidney disease (ESKD), and even death (KDIGO, 2024). Diabetes, hypertension, autoimmune diseases and genetic susceptibility are all major causes CKD. Its pathological process involves complex signaling pathways, such as inflammatory responses, oxidative stress, apoptosis and fibrosis (Romagnani et al., 2017; Burnier and Damianaki, 2023). Although various clinical interventions are available, effectively halting CKD progression remains challenging to date (Romagnani et al., 2025). There is an urgent need to delve into its molecular mechanism to find new therapeutic targets.

Post-translational modification (PTM) of proteins is a chemical modification process after the completion of protein translation, which can regulate the activity, stability, localization and intermolecular interactions of proteins (Wang et al., 2022). A growing number of research findings suggest PTMs plays an important role in the pathophysiological process of CKD (Noels et al., 2024), especially in diabetic nephropathy (DN), where modifications such as protein deubiquitinating modification affect the process of podocyte inflammation and injury (Zhao et al., 2024b). PTMs have been reported to be classified into two types: non-enzymatic PTMs and enzymatic PTMs (ePTMs) (Jennings et al., 2022). Non-enzymatic PTM is usually triggered by the direct reaction of proteins with active metabolites (Tang and Kalim, 2022). In CKD, oxidative stress and metabolic disorders accelerate a significant increase in non-enzymatic PTMs, intensifying the inflammatory response and fibrosis in renal tissue (Noels et al., 2024). Taking the advanced glycation end products as an example, they can activate downstream signaling pathways such as nuclear factor-kappa B (NF-κB) and mitogen-activated protein kinase (MAPK) by binding to the cell surface receptor, subsequently leading to the release of pro-inflammatory cytokines (Stinghen et al., 2016; Wang and Zhang, 2024).

EPTMs rely on the catalysis of specific enzymes and are characterized by strong reversibility and precise regulation (Li Z. et al., 2025). Here, nine key ePTMs are highlighted, including methylation, acetylation, ubiquitination, glycosylation, lactylation, palmitoylation, crotonylation, small ubiquitin-like modifier (SUMO)-mediated modification, and prenylation. They have received increasing attention in CKD, participating in core pathological processes such as fibrosis and inflammation, and showing potential as therapeutic targets (Laget et al., 2022). However, their mechanisms of action have not yet been systematically integrated. Notably, phosphorylation has been extensively studied in chronic kidney disease with many established databases (e.g., PhosphoSitePlus, and PhosphoGRID), so it is not listed separately here to avoid redundancy, but will be mentioned when it crosstalks with other ePTMs (Li K. et al., 2024; Šakić et al., 2024; Li J. et al., 2024). Accumulating evidence indicates these ePTMs are not merely passive markers of CKD progression (e.g., Gd-IgA1), but actively participate in disease etiology by regulating core pathological processes (Vaz de Castro et al., 2024; Li J. et al., 2024). For example, histone deacetylases (HDACs) regulate histone acetylation and deacetylation, and their inhibitors (such as vorinostat and romidepsin) have been approved by the U.S. Food and Drug Administration (FDA) for treating lymphoma. (Mabe et al., 2024; Roza et al., 2023). Recent studies have also explored inhibitors or activators targeting protein kinases, methyltransferases, and ubiquitin ligases to modulate ePTMs in disease treatment (Mabe et al., 2024). The development of such drugs requires a thorough understanding of these enzymes’ structure, function and roles in CKD.

Given the unique advantages of ePTMs in disease regulation, this review focuses on three key aspects: (1) the molecular mechanisms of ePTMs and their regulatory enzymes (e.g., acetyltransferases and deacetylases); (2) the roles of ePTMs in the development and progression of CKD, including tubulointerstitial fibrosis, inflammation, and metabolic disturbances; (3) the potential of ePTMs as diagnostic markers and therapeutic targets for CKD, with particular attention to current drug development and future directions in precision medicine. This review aims to bridge the gap between basic research and clinical application, laying the groundwork for the development of more effective therapies for CKD.

## 2 Methylation

### 2.1 Enzymatic mechanisms of methylation

Protein methylation is a major form of ePTMs involved in the pathogenesis of CKD (Noels et al., 2024). It entails the enzymatic transfer of a methyl group (–CH_3_) to specific amino acids, mainly lysine and arginine, in forms such as monomethylation (me1), dimethylation (me2), and trimethylation (me3). Lysine may undergo me1, me2, or me3, while arginine can be modified by me1, symmetric dimethylation (me2s), or asymmetric dimethylation (me2a) (Paik et al., 2007; Jin et al., 2023b). These modifications regulate protein function, interactions, and crosstalk with other PTMs, thus influencing various physiological and pathological processes (Jin et al., 2023b; Tan et al., 2024).

**FIGURE 1:**
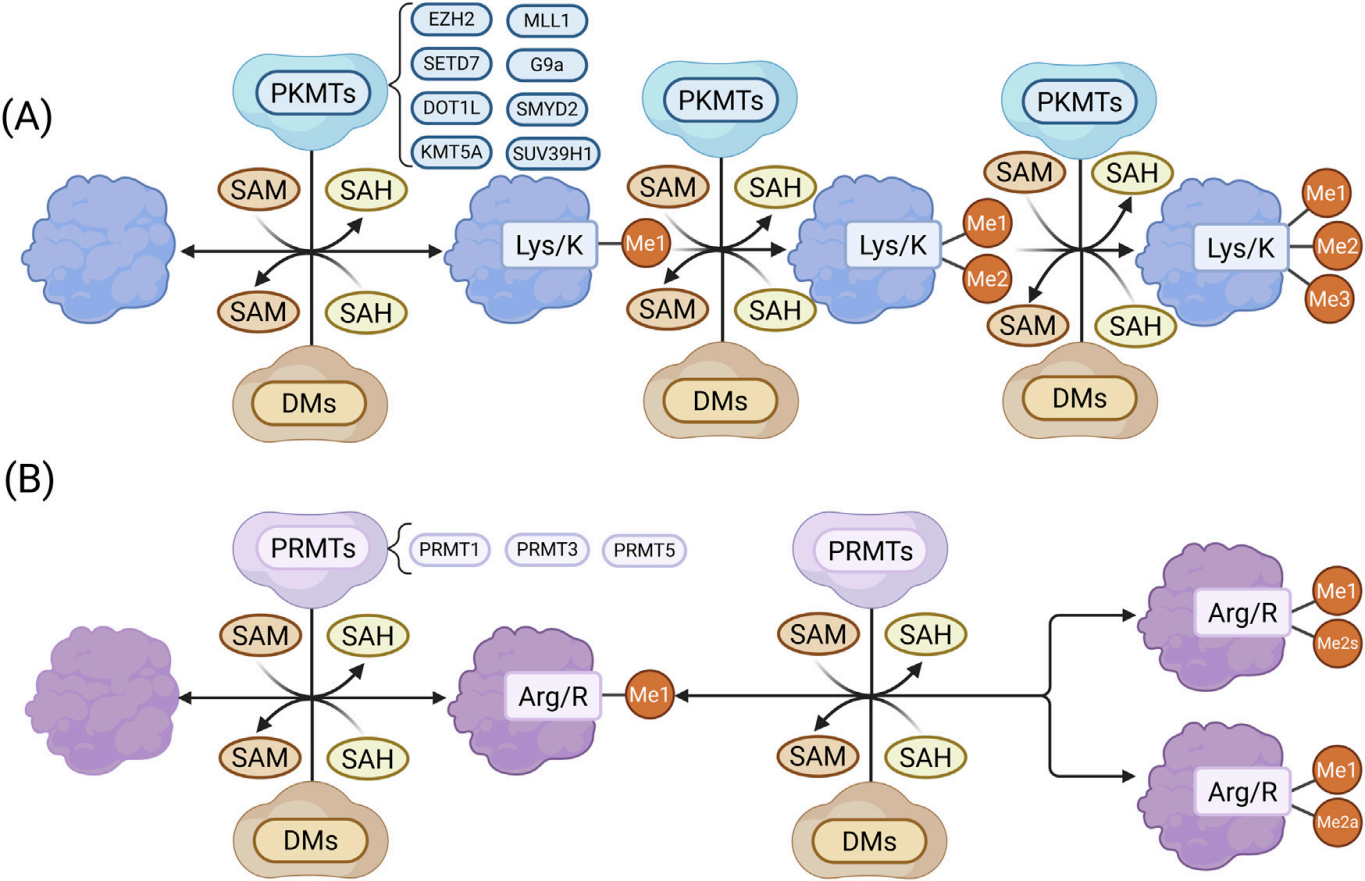
**(A)** Lysine methylation: PKMTs catalyze the transfer of a methyl group from SAM to lysine residues, generating me1 and SAH. The process may continue to form me2 and me3 proteins. **(B)** Arginine methylation: PRMTs transfer a methyl group from SAM to arginine residues, producing me1 proteins and SAH, followed by either me2s or me2a. Methylated proteins can be reversed by demethylases (DEs). (Created in https://BioRender.com).

Protein lysine methyltransferases (PKMTs) and protein arginine methyltransferases (PRMTs) are the main enzymes responsible for methylation (Luo, 2018; Wu et al., 2021). Using S-adenosylmethionine (SAM) as a methyl donor, they catalyze methylation via a bimolecular nucleophilic substitution reaction, generating methylated proteins and S-adenosylhomocysteine (SAH). Over 50 PKMTs have been identified, many of which target histone and non-histone proteins (Schnee et al., 2024). The largest subgroup, Su(var)3-9, Enhancer-of-zeste, Trithorax (SET) domain, containing PKMTs, share a conserved domain that binds both substrate and SAM (Schnee et al., 2024). For instance, (Su(var)3-9, enhancer of zeste, Trithorax) domain-containing protein 7 (SETD7) and enhancer of zeste homolog 2 (EZH2) catalyze methylation at histone H3 lysine 4 (H3K4) and lysine 27 (H3K27), respectively (Sun et al., 2010; Su et al., 2022). PRMT1-9 are the known arginine methyltransferases, with PRMT1 generating asymmetric dimethylarginine, a contributor to endothelial dysfunction in CKD (Figure 1).

Histone methylation regulates gene expression by altering chromatin structure. H3K4me3, mediated by SETD7, promotes transcription of pro-fibrotic genes like collagen1alpha (Col1α1) and connective tissue growth factor (CTGF) (Sun et al., 2010), whereas H3K27me3, catalyzed by EZH2, suppresses anti-fibrotic gene expression (Wu et al., 2024). Methylation of non-histone proteins, such as transcription 3 (STAT3) and nuclear factor-kappa B (NF-κB) p65, also affects signaling and cell cycle regulation (Li L. X. et al., 2017; Cui et al., 2022). Overall, protein methylation contributes to CKD by modulating gene expression and cellular function, offering potential therapeutic targets. Further studies are needed to clarify its roles and clinical applications in CKD.

### 2.2 Effect of methylation in the pathological progression of CKD

In CKD, histone methylation modulates gene transcription and contributes to disease progression (Table 1). The histone methyltransferase EZH2 catalyzes H3K27 trimethylation. Elevated levels of the long non-coding RNA (lncRNA) antisense non-coding RNA in the INK4 locus (ANRIL) in CKD recruit EZH2 to the brain-derived neurotrophic factor (BDNF) promoter, reducing BDNF expression and leading to endothelial dysfunction and mitochondrial imbalance (Su et al., 2022). In diabetic nephropathy (DN), lncRNA Dlx6os1 enhances EZH2-mediated H3K27me3 at the SRY-box transcription factor 6 (SOX6) promoter, promoting mesangial cell proliferation, fibrosis, and inflammation (Chen Y. X. et al., 2022). In the ischemia/reperfusion (I/R) and folic acid (FA)-induced acute kidney injury to chronic kidney disease (AKI-to-CKD) transition models, EZH2 can induce trimethylation of histone H3 and represses phosphatase and tensin homolog (PTEN) expression, subsequently activating epidermal growth factor receptor (EGFR)/ERK1/2/STAT3 signaling to drive epithelial–mesenchymal transition (EMT) and G2/M arrest, thereby mediating the progression from acute kidney injury to chronic kidney disease (Zhou et al., 2023). So, inhibiting EZH2 preserves mothers against decapentaplegic homolog (Smad7) and PTEN levels, blocking transforming growth factor beta (TGF-β)/Smad3, EGFR, and platelet-derived growth factor receptor beta (PDGFRβ) signaling and thereby alleviating fibrosis (Zhou et al., 2016). However, It has also been shown that long noncoding (lnc)RNA AFAP1-AS1 can interact with EZH2 to upregulate the level of H3K27me3 in the NOD-like receptor protein 3 (NLRP3) promoter region in M2 macrophage-derived exosomes, which inhibits the protein levels of NLRP3, cleaved caspase-1, gasdermin D (GSDMD)-N, as well as the levels of interleukin (IL)-18, IL-1β, and lactate dehydrogenase (LDH), and ultimately suppresses podocyte pyroptosis (Zhan et al., 2025).

**TABLE 1:** Regulatory networks and pathological effects of PKMTs and PRMTs in CKD.

Involved methyltransferases	Involved proteins and modification change	Pharmacological effects	Involved pathways	Pathological model	References
EZH2	H3K27 ↑	Activates PERK and ATF6, promotes ER stress and EMT; regulates BDNF via EZH2-mediated H3K27me3	TGF-β pathway PI3K-Akt pathway	ANCA-associated patient samples CKD mouse models	Su et al. (2022)
		Represses SOX6 via recruiting EZH2, promotes fibrosis and inflammation	TGF-β/Smad pathway NF-κB pathway	DN mice Streptozotocin (STZ)-induced DN mice HG-treated SV40 MES13 cells	Chen et al. (2022b)
		Promotes PTEN repression, activates EGFR/ERK1/2/STAT3 and TGF-β/Smad3 pathways; induces M2 macrophage polarization via STAT6 and PI3K/AKT	EGFR/ERK1/2/STAT3 pathway PI3K/Akt pathway	FA-induced mouse model H_2_O_2_-treated Hexokinase2 cells	Zhou et al. (2023)
		Suppresses Smad7 and PTEN, activates TGF-β/Smad3, EGFR/PDGFRβ/STAT3/ERK1/2 pathways	TGF-β/Smad3 pathway, EGFR/PDGFRβ/STAT3/ERK1/2 pathway	UUO mouse model, serum/TGF-β1-stimulated renal fibroblasts	Wang et al. (2018)
EZH2	NLRP3 ↑	In M2 macrophage-derived exosomes, lncRNA AFAP1-AS1 expression is upregulated, and it interacts with EZH2 to upregulate the level of H3K27me3 in the NLRP3 promoter region, thereby inhibiting the protein levels of NLRP3, Cleaved caspase-1, GSDMD-N, to suppress podocyte pyroptosis	NLRP3/Caspase-1/IL-18/IL-1β pathway	high glucose induced podocyte injury model	Zhan et al. (2025)
MLL1	H3K4 ↑	Suppresses lncZEB1-AS1, downregulates ZEB1 via H3K4me3 modification	p53/lnc ZEB1-AS1/ZEB1 pathway	db/db mice STZ-induced DN mice HG-treated HK-2 cells, human DN patient biopsies	Wang et al. (2018)
		Promotes EMT (α-SMA, fibronectin), activates TGF-β/Smad3 and AKT pathways, regulates H3K4me1	TGF-β/Smad pathway	UUO mice TGF-β1-stimulated renal cells	Zou et al. (2023)
SETD7	H3K4 ↑	Promotes ECM gene expression (Col1α1, CTGF, PAI-1), regulates TGF-β1-induced H3K4me1	TGF-β/Smad pathway	HG-stimulated mesangial cells UUO mice	Sun et al. (2010)
		Inhibits α-SMA and fibronectin, reduces H3K4me1, preserves klotho expression	TGF-β/Smad pathway	UUO mice TGF-β1-stimulated renal cells	Sasaki et al. (2016)
G9a	H3K9 ↑	Promotes H3K9me1, represses klotho, activates TGF-β/Smad3 pathway, induces EMT (α-SMA, collagen-1)	TGF-β/Smad3 pathway	UUO mice, human IgA nephropathy and membranous nephropathy biopsy samples	Irifuku et al. (2016)
		Recruits G9a to Klotho promoter, increases H3K9me1, represses Klotho transcription, activates TGF-β/Smad3 and AKT pathways, induces endothelial-to-mesenchymal transition (α-SMA, vimentin, COL I, COL III)	TGF-β/Smad3 and AKT pathway	DN (human and rat models) HG-induced Human Umbilical Vein Endothelial Cells (HUVECs) injury	Li et al. (2019)
SUV39H1	H3K9 ↓	Decreases H3K9me3 at inflammatory gene promoters (IL-6, MCSF, MCP-1), reduces recruitment of HP1, activates NF-κB pathway, enhances TNF-α-induced inflammatory response	NF-κB pathway	Diabetic db/db mice HG-treated human vascular smooth muscle cells (VSMCs)	Villeneuve et al. (2008)
DOT1L	H3K79 ↓	Interacts with HDAC2, modulates H3K79me2 and H3 acetylation at Edn1 promoter, upregulates ET1, activates TGF-β/Smad3 pathway, induces renal fibrosis	TGF-β/Smad3 pathway	UUO mice STZ-induced diabetic mice	Zhang et al. (2020a)
PRMT1	H4R3 ↑	Activates PERK and ATF6, promotes ER stress and EMT; regulates glycolysis via HIF-1α	Activate PERK and ATF6	DN mouse model Hexokinase2 cells	Chen et al. (2019)
JMJD3	H3K27me2 ↑	JMJD3 is upregulated and associated with increased H3K27 dimethylation. It promotes myeloid fibroblast activation and M2 macrophage polarization by inducing IRF4	JMJD3-IRF4-M2 pathway	UUO-induced mice	An et al. (2023)
PRMT3	HIF-1α ↑	Methylates HIF-1α, promotes glycolysis and VC	HIF-1α pathway	CKD mouse model and VSMCs	Zhou et al. (2024a)
PRMT5	SREBP1 ↑	Interacts with LINC01138 to enhance SREBP1 methylation and stability, promotes lipid desaturation	Fatty acid desaturation pathway	ccRCC cell lines and patient samples	Zhang et al. (2018)
	H3R8 ↓	HDAC3 and PRMT5 maintain the basal repressed state of TGF-β target gene Smad7	TGF-β/Smad pathway	HeLa cells HepG2 cells	Tabata et al. (2009)
	p65 ↑	Promotes binding of p65 to DNA, activates NF-κB pathway, regulates expression of inflammatory factors like IL-6 and TNF-α	NF-κB pathway	IL-1β-stimulated 293IL1R cell model	Wei et al. (2013)
SMYD2	STAT3 ↑, p65 ↑	Activates STAT3 and NF-κB pathways, promotes secretion of inflammatory factors such as IL-6/TNF-α; inhibits p53 phosphorylation, regulates BAX/BCL-2 ratio to affect apoptosis; regulates proliferating cell nuclear antigen, Cyclin D1, and p21 expression to influence cell proliferation	JAK/STAT pathway NF-κB pathway	Autosomal dominant polycystic kidney disease (ADPKD) model	Li et al. (2017a)
KMT5A	H4K20 ↓	Downregulates KMT5A, increases RFX1 binding to ENO1 promoter, upregulates ENO1, activates TGF-β/Smad3 pathway, induces endothelial-to-mesenchymal transition (α-SMA, vimentin, COL I, COL III)	TGF-β/Smad3 pathway	DN (human and rat models) HG-treated HUVECs	Lu et al. (2021)

The target protein modification level change refers to the comparison between normal physiological conditions and CKD, pathological state. ↑ indicates an increase in the level of epigenetic modifications. ↓ indicates a decrease in the level of epigenetic modifications.

Mixed lineage leukemia 1 (MLL1), another histone methyltransferase, interacts with zinc finger E-box binding homeobox 1 antisense 1 (ZEB1-AS1) in DN to enhance H3K4me3 at the ZEB1 promoter, exerting anti-fibrotic effects (Wang et al., 2018). In unilateral ureteral obstruction (UUO) model, MLL1 and its cofactor menin increase H3K4me1 and activate transforming growth factor-beta1 (TGF-β1) signaling, inducing EMT-related transcription factors and fibrotic markers (Zou et al., 2023). High glucose (HG) also enhances H3K4 methylation at extracellular matrix (ECM) gene promoters via TGF-β1 signaling, promoting ECM production (Sun et al., 2010). TGF-β1 upregulates SET7/9 through Smad3, increasing H3K4me1 and activating pro-fibrotic genes (Sasaki et al., 2016). It also induces G9a, which raises H3K9me1 and suppresses the anti-fibrotic gene Klotho (Irifuku et al., 2016). LncRNA metastasis-associated lung adenocarcinoma transcript 1 (MALAT1) recruits G9a to the Klotho promoter, further reducing its expression and contributing to HG-induced endothelial injury (Li et al., 2019). In DN, reduced of the suppressor of variegation 3-9 homolog 1 (SUV39H1) expression and lower H3K9me3 levels correlate with elevated inflammatory gene expression (Villeneuve et al., 2008). Disruptor of Telomeric Silencing 1-Like (DOT1L), which catalyzes H3K79me2, represses Endothelin 1 (EDN1) transcription; its deficiency upregulates EDN1 and promotes fibrosis (Zhang L. et al., 2020). Jumonji domain-containing protein-3 (JMJD3), a histone H3K27 demethylase, is significantly increased with elevated H3K27 dimethylation, promotes myeloid fibroblast activation and M2 macrophage polarization via the JMJD3-IRF4 axis in UUO mice, and this can be reversed by the JMJD3 inhibitor GSK-J4 (An et al., 2023).

Non-histone methylation also contributes to CKD pathogenesis. In DN, high glucose upregulates PRMT1, which mediates H4R3me2a modification at the activating transcription factor 6 (ATF6) promoter, activating the protein kinase R-like endoplasmic reticulum kinase (PERK) and ATF6 pathways and triggering the endoplasmic reticulum (ER) stress and apoptosis in tubular cells (Chen et al., 2019). PRMT1 also deposits H4R3me2 at the ATF6 promoter, inducing EMT and fibrosis (Chen et al., 2019). In CKD, PRMT3 stabilizes the transcription factor hypoxia-inducible factor-1alpha (HIF-1α) via methylation, enhancing glycolysis and promoting vascular smooth muscle cell (VSMC) osteogenic transformation and vascular calcification (VC) (Zhou G. et al., 2024). PRMT5 interacts with long intergenic non-coding RNA located on 1q21.2 (LINC01138) in renal carcinoma to induce Sterol regulatory element-binding protein 1 (SREBP1) me2s, supporting lipid synthesis and tumor proliferation (Zhang et al., 2018). It also mediates basal repression of the Samd7 promoter and enhances NF-κB signaling under IL-1β stimulation by catalyzing R30 dimethylation of p65 (Tabata et al., 2009; Wei et al., 2013).

SET and MYND domain-containing protein 2 (SMYD2) methylates p53 at K370, suppressing its transcriptional activity and promoting cyst epithelial cell survival (Li L. X. et al., 2017). It also modifies STAT3 and p65, activating genes linked to inflammation and proliferation (Li L. X. et al., 2017). In DN, HG reduces lysine methyltransferase 5A (KMT5A) expression, lowering H4K20me1 and lifting transcriptional repression of Enolase 1 (ENO1), which contributes to endothelial–mesenchymal transition and fibrosis (Lu et al., 2021). Collectively, histone and non-histone methylation regulate gene expression, signaling pathways, and phenotypic transitions, playing central roles in CKD development.

### 2.3 The therapeutic potential of methylation in CKD

Given the role of methylation in CKD progression, methyltransferase inhibitors show therapeutic potential by modulating histone and non-histone methylation (Table 2). The EZH2 inhibitor 3-deazaneplanocin A (3-DZNeP) attenuates fibrosis in UUO model by suppressing TGF-β/Smad3 and EGFR/PDGFR signaling, thereby reducing fibroblast activation and ECM deposition (Zhou et al., 2016). SET7/9 inhibitors such as sinefungin and the selective inhibitor PFI-2 reduce H3K4me1 levels and inhibit fibrosis. Sinefungin blocks TGF-β1-induced fibroblast activation and ECM production (e.g., α-SMA, collagen I) (Sasaki et al., 2016), while PFI-2 suppresses Th2 cytokines (IL-4, IL-13), inhibits M2-to-myofibroblast transition, and reduces nuclear NF-κB p65 translocation (Liu et al., 2021). The DOT1L inhibitor EPZ5676 decreases H3K79me2, stabilizes PTEN and Smad7, and suppresses EMT and fibroblast activation (Liu L. et al., 2019). PRMT1 inhibitor AMI-1 and G9a inhibitor BIX01294 lower H4R3me2a and H3K9me1 levels, respectively, thereby inhibiting Smad3 phosphorylation and TGF-β1-induced fibrosis (Zhu Y. et al., 2020; Irifuku et al., 2016). MLL1 complex inhibitors MI-503 and MM102 also reduce renal injury (Zhang et al., 2022; Zou et al., 2023). Additionally, the demethylase inhibitor GSK-J4 prevents H3K27 demethylation by targeting KDM6A, downregulating DKK1 and TGF-β1, and alleviating fibrosis in DN (Hung et al., 2022). Furthermore, methyltransferase inhibitors have also been shown to alleviate CKD-related complications. For example, PRMT3 inhibitor SGC707 reduces arginine methylation of HIF-1α and attenuates VC (Zhou G. et al., 2024). The SMYD2 inhibitor AZ505 lowers methylation of STAT3, NF-κB p65, and H3K36me3, thereby reducing tubular cell apoptosis and inflammation (Li L. X. et al., 2017; Cui et al., 2022). In general, these compounds targeting methyltransferases regulate histone and non-histone methylation through various signaling pathways such as TGF-β/Smad3 and EGFR/ERK1/2/STAT3, and exert ameliorative effects in pathological conditions of CKD such as renal fibrosis. This indicates that methylation is a driver of the progression of CKD and its complications, and regulating its related enzymes could be a potential research direction for CKD treatment. It should also be noted that most compounds that regulate methylation are still in preclinical stages such as laboratory or animal studies, and there is a long development process ahead before they can be practically applied in clinic.

**TABLE 2:** Small molecules targeting methylation modifications in CKD and their target proteins.

Small molecule	Targets	Phase	Experiental models	References
3-DZNeP	EZH2	Preclinical study	UUO induced renal fibrosis mouse model TGF-β1-induced NRK-49F TGF-β1-induced Renal Proximal Tubular Cells (RPTCs)	Zhou et al. (2016)
Sinefungin	SET7/9	Preclinical study	UUO induced renal fibrosis mouse model TGF-β1-induced NRK-49F TGF-β1-induced NRK-52E	Sasaki et al. (2016)
PFI-2	SETD7	Preclinical study	FA-induced renal fibrosis mouse model UUO induced renal fibrosis mouse model Myofibroblast Activation Model	Liu et al. (2021)
EPZ5676	DOT1L	Phase 1b/2	UUO induced renal fibrosis mouse model TGF-β1-induced NRK-49F TGF-β1-induced RPTCs	Liu et al. (2019b)
AMI-1	PRMT1	Preclinical study	UUO induced renal fibrosis mouse model TGF-β1-induced NRK-49F TGF-β1-induced RPTCs	Zhu et al. (2020b)
SGC707	PRMT3	Preclinical study	High-phosphate diet-induced CKD mouse model β-glycerophosphate-induced VSMCs	Zhou et al. (2024a)
BIX01294	G9a	Preclinical study	UUO induced renal fibrosis mouse model TGF-β1-induced NRK-52E TGF-β1-induced HK-2 cells	Irifuku et al. (2016)
AZ505	SMYD2	Preclinical study	PKD1-knockout polycystic kidney mouse model mIMCD3 cells	Li et al. (2017a)
MI-503	MLL1-menin	Preclinical study	UUO induced renal fibrosis mouse model TGF-β1-induced RPTCs TGF-β1-induced NRK-49F	Zou et al. (2023)
GSK-J4	KDM6A	Preclinical study	STZ -induced diabetic mouse model HG-induced RMC model	Hung et al. (2022)

The Phase data for small molecules are derived from the Chinese Clinical Trial Registry (https://www.chictr.org.cn/index.html), the Drug Clinical Trial Registration and Information Disclosure Platform (http://www.chinadrugtrials.org.cn), ClinicalTrials.gov (https://clinicaltrials.gov), the ICTRP Search Portal (https://trialsearch.who.int/), and the official website of the National Medical Products Administration (https://www.nmpa.gov.cn).

## 3 Acetylation

### 3.1 Acetylation modification and its key enzymes involved

Acetylation is a reversible modification in which an acetyl group is transferred from acetyl-coenzyme A (CoA) to the N-terminus or ε-amino group of lysine residues. In CKD, widespread protein acetylation has been detected in renal tubular epithelial cells and contributes to disease progression (Pan et al., 2024; Tan et al., 2024). A proteomic study using immunoaffinity enrichment and liquid chromatography-mass spectrometry identified 2,012 acetylated proteins and 4,311 acetylation sites in mouse tubular epithelial cells under high glucose conditions (Wan et al., 2021), with most located in the mitochondria, nucleus, and cytoplasm.

**FIGURE 2:**
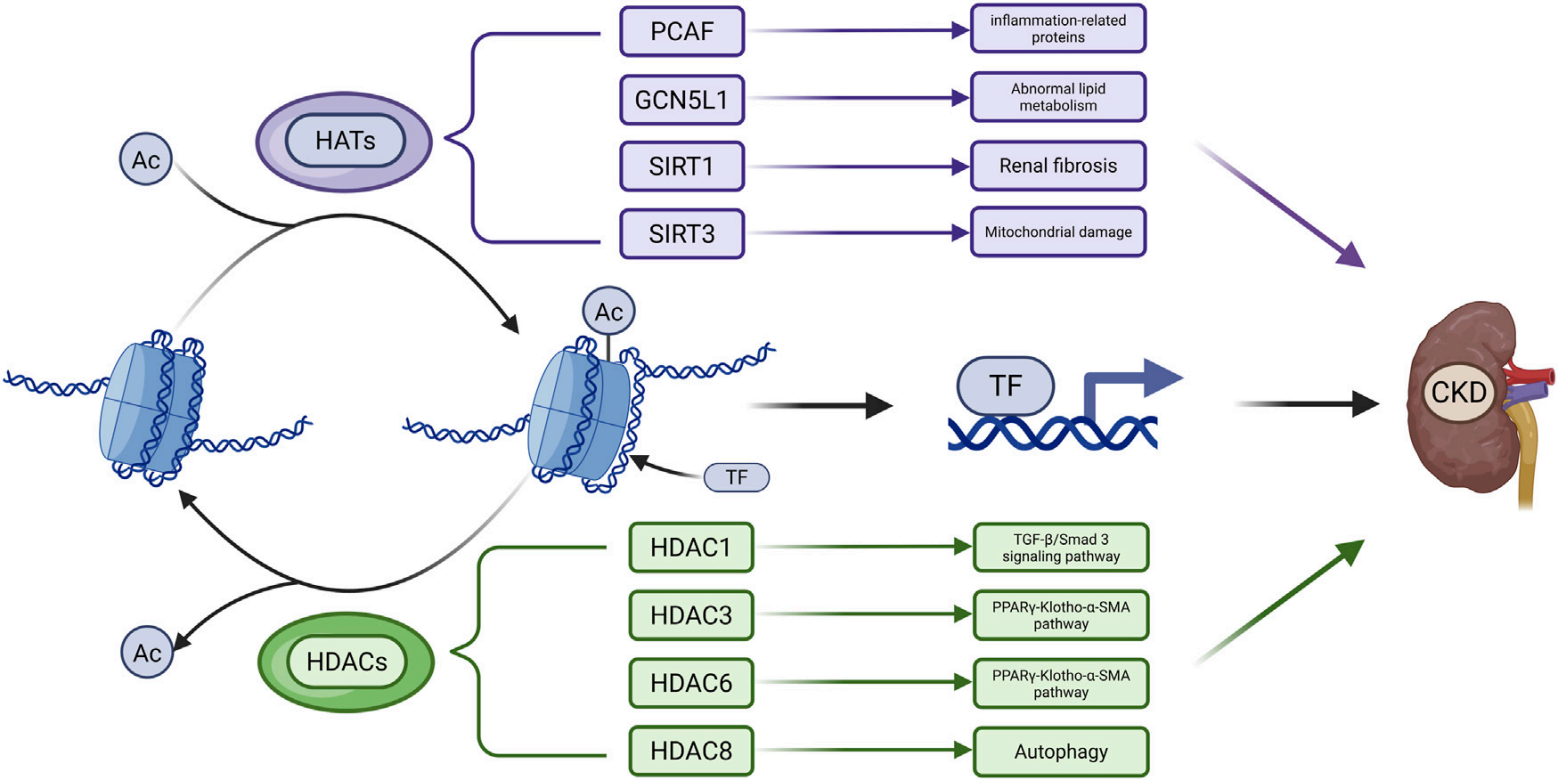
Main Mechanisms of Acetylation in CKD. Acetyl groups (Ac) are transferred to histone amino acid residues by HATs, leading to a more relaxed chromatin structure. This facilitates the binding of transcription factors (TFs) to DNA and thereby regulates gene expression. Acetylated proteins can be deacetylated by HDACs. The dysregulation of acetylation and deacetylation processes contributes to the accelerated progression of CKD. (Created in https://BioRender.com).

Histones are among the most extensively acetylated proteins. Their acetylation is regulated by histone acetyltransferases (HATs), which add acetyl groups, and HDACs, which remove them (Figure 2). HATs, or “writers,” catalyze lysine acetylation, neutralizing histone charge, loosening chromatin, and promoting transcription by enhancing DNA accessibility (Singh et al., 2024). HATs are classified into three main families: p300/CREB-binding protein (CBP), MYST (e.g., Esa1, Sas2, Tip60, MOF, MOZ, MOR), and GCN5-related N-acetyltransferase (GNAT) (e.g., GCN5, PCAF, Elp3, Hpa2, Hat1) (White et al., 2024). HDACs, or “erasers,” remove acetyl groups, leading to chromatin condensation and transcriptional repression (Seto and Yoshida, 2014). Eighteen HDACs have been identified and are grouped into four classes based on structure and localization: Class I (HDAC1, 2, 3, 8), Class IIa (HDAC4, 5, 7, 9), Class IIb (HDAC6, 10), Class III (sirtuins, SIRT1–7), and Class IV (HDAC11) (Singh et al., 2024).

### 3.2 Effect of acetylation in the pathological progression of CKD

In CKD, an imbalance between HATs and HDACs disrupts gene regulation and accelerates disease progression (Table 3). While acetylation levels remain low in healthy kidneys, they are markedly increased in fibrotic renal tissue (Zhang et al., 2021b). Among HATs, acetyltransferase p300/CBP-associated factor (PCAF) and General control of amino acid synthesis 5 like 1 (GCN5L1) are implicated in CKD. PCAF is upregulated in the kidneys of db/db and lipopolysaccharide-treated mice, where it increases histone acetylation (e.g., H3K18ac) and activates inflammatory gene promoters such as Intercellular adhesion molecule-1 (ICAM-1) (Huang J. et al., 2015). GCN5L1 is highly expressed in kidneys of high-fat diet (HFD)-fed mice and regulates acetylation of fatty acid oxidation enzymes, including long-chain acyl coenzyme A dehydrogenase (LCAD) and beta-hydroxyacyl-CoA dehydrogenase (β-HAD), thereby modulating their activity (Lv et al., 2019).

**TABLE 3:** Regulatory networks and pathological effects of HATs and HDACs in CKD.

Involved enzymes	Involved Proteins and modification change	Pharmacological effects	Involved pathways	Pathological model	References
PCAF	H3K18ac ↑, H3K9ac ↑	PCAF-mediated histone acetylation primarily romoted inflammatory responses by upregulating the expression of inflammation-related proteins such as ICAM-1, VCAM-1, MCP-1, and NF-κB p50	NF-κB pathway	Db/db mouse model Lipopolysaccharide-induced mouse model	Huang et al. (2015a)
GCN5L1	LCAD ↑ β-HAD ↑	High expression of GCN5L1 promotes acetylation modification of LCAD and β-HAD, inhibits mitochondrial fatty acid oxidation rate, causes intracellular accumulation of triglycerides and acyl-CoA, induces renal toxicity and EMT, and promotes the progression of renal fibrosis	TGF-β/Smad pathway	HFD-induced mouse model PA-induced HK-2 cells	Lv et al. (2019)
SIRT1	Smad3 ↑	Low expression of SIRT1 leads to increased acetylation level of Smad3, and promotes the expression of TGF-β target genes, and drives the progression of renal fibrosis	TGF-β/Smad3 pathway	5/6 Nephrectomy (Nx) rat model 5/6 Nx mouse model MES-13 cells	Huang et al. (2014)
	p53 ↑	Low expression of Sirt1 leads to increased acetylation level of p53, which in turn promotes the expression of pro-apoptotic protein Bax and fibrosis-related proteins α-SMA and Fibronectin, exacerbating renal interstitial fibrosis	p53/SIRT1/NF-κB signal axis	UUO-induced mouse model Hypoxia/serum deprivation (H/SD) HUVECs	Wang et al. (2020b)
	PERK ↑	Reduced Sirt1 expression leads to increased acetylation of PERK at lysine K889, activating the PERK-eIF2α-ATF4 ER stress pathway to drive the progression of VC.	PERK/eIF2α/ATF4 ER stress pathway	Adenine-induced mouse model β-Glycerophosphate-induced VSMCs	Zhang et al. (2021a)
SIRT3	PDHE1α ↑, CPT1α ↑, ATP5O ↑	Reduced expression of SIRT3 in CKD leads to increased acetylation levels of mitochondrial proteins PDHE1α, CPT1a, and ATP5O. This process impairs mitochondrial energy metabolic pathways, including the tricarboxylic acid cycle, fatty acid β-oxidation, and oxidative phosphorylation, thereby promoting the progression of renal fibrosis	Mitochondrial energy metabolism pathway	Sirt3 knockout mice UUO mouse model TGF-β1-induced TECs	Zhang et al. (2021b)
					Xiong et al. (2025b)
HDAC	H3 ↓	HDAC suppressed the transcription of the Klotho gene through deacetylation of histone H3, leading to imbalance of mineral metabolism-related factors such as FGF23 and PTH, and simultaneously activates the TGF-β/Smad3 signaling pathway, promoting renal interstitial fibrosis and bone remodeling abnormalities	TGF-β/Smad3 pathway	Adenine-induced chronic kidney disease-mineral and bone disorder model Trichostatin A-induced HK-2 cells	Lin et al. (2017a)
HDAC1	DUSP1 ↓	HDAC1 promotes the deacetylation of DUSP1, inhibits the expression of DUSP1, weakens its dephosphorylation effect on Smad3, leads to the excessive phosphorylation of Smad3 at the 423/425 sites and its nuclear translocation, activates the TGF-β/Smad3 signaling pathway, and finally promotes the progression of renal fibrosis	TGF-β/Smad3 pathway	UUO-induced mouse model TGF-β1-induced HK-2 cells	Wang et al. (2025)
HDAC3	PPARγ ↓	HDAC3 promotes the deacetylation of PPARγ, inhibits the transcriptional activity of PPARγ, leads to the downregulation of Klotho expression, and promotes the expression of renal fibrosis-related proteins (such as α-SMA)	PPARγ/Klotho pathway TGF-β/Smad pathway	Adenine-induced mouse model TGF-β1-induced HK-2 cells	Lin et al. (2017b)
HDAC6	TFEB ↓	HDAC6 promotes the deacetylation of TFEB, inhibits the nuclear translocation and transcriptional activity of TFEB, leads to the downregulation of key genes (such as beclin 1 and ATG7) in the autophagy-lysosome pathway, and promotes cell death and renal interstitial fibrosis	HDAC6/TFEB autophagy regulatory pathway	5/6 SNx rat model Thapsigargin-induced NRK-52E cells	Brijmohan et al. (2018)
HDAC8	Cortactin ↓	HDAC8 promotes the deacetylation of cortactin, activates the TGF-β1/Smad3, STAT3 and β-catenin signaling pathways, induces renal tubular epithelial cells to arrest in the G2/M phase, upregulates the transcription factor Snail, and downregulates BMP7 and Klotho	TGF-β1/Smad3 pathway JAK/STAT3 pathway Wnt/β-catenin pathway	UUO-induced mouse model	Zhang et al. (2020b)

The target protein modification level change refers to the comparison between normal physiological conditions and CKD, pathological state. ↑ indicates an increase in the level of epigenetic modifications. ↓ indicates a decrease in the level of epigenetic modifications.

HDACs such as SIRT1, SIRT3, HDAC1, HDAC3, HDAC6, and HDAC8 contribute to CKD pathogenesis by modulating histone and non-histone acetylation. SIRT1 knockout in the ischemia-reperfusion injury (IRI) model exacerbates renal injury and fibrosis by inhibiting H3K27 acetylation at the ATP citrate lyase (ACLY) promoter to impair fatty acid oxidation (FAO), binding to and deacetylating Smad3 to enhance its transcriptional activity, and deacetylating p53 at K382 and K320 to suppress apoptosis (Huang et al., 2014; Wang Y. et al., 2020). SIRT1 overexpression improves renal function, while acetylation at its K889 site regulates PERK activity, reduces ER stress, and mitigates VC (Zhang et al., 2021a). SIRT3 prevents hyperacetylation of pyruvate dehydrogenase (PDH), E1α, carnitine palmitoyl-transferase 1 A (CPT1A), and ATP synthase subunit O (ATP5O) in UUO and Sirt3-deficient mice (Zhang et al., 2021b). Acetylation at pyruvate dehydrogenase 1alpha (PDHE1α) K385 is essential for PDH function under pro-fibrotic stress (Zhang et al., 2021b). Several HDACs suppress protective gene expression. In adenine-fed mice, HDACs reduce Klotho expression by removing H3K9 acetylation. In UUO model, HDACs inhibit dual specificity phosphatase 1 (DUSP1), which is associated with renal dysfunction and fibrosis via Smad3 activation (Lin et al., 2017a; Wang et al., 2025). HDAC3 inhibition enhances Klotho expression by promoting peroxisome proliferator-activated receptor gamma (PPARγ) acetylation and DNA binding (Lin et al., 2017b), while HDAC6 inhibition promotes TFEB acetylation and nuclear translocation, activating autophagy (Brijmohan et al., 2018). TF3, which co-localizes with H3K27Ac, may preserve acetylation by recruiting CBP/p300 (Yang et al., 2025). HDAC1 also regulates DUSP1, and its loss contributes to fibrosis through Smad3 signaling (Wang et al., 2025). HDAC8 is upregulated in UUO and deacetylates cortactin, thereby activating TGF-β1/Smad3, STAT3, and β-catenin pathways while suppressing bone morphogenetic protein 7 (BMP7) and Klotho to promote interstitial fibrosis (Zhang Y. et al., 2020). In conclusion, HATs and HDACs have diverse and complex roles in CKD progression. Their targeted regulation may offer promising strategies for therapeutic intervention.

### 3.3 The therapeutic potential of acetylation in CKD

Several pharmacological agents targeting acetylation have demonstrated therapeutic potential in CKD models (Table 4). Trichostatin A, an HDAC1/2/3 inhibitor, alleviates fibrosis and mineral metabolism disorders in UUO and adenine-induced models by increasing histone and PPARγ acetylation, suppressing colony-stimulating factor-1 (CSF-1), and upregulating Klotho (Marumo et al., 2010; Azechi et al., 2013; Lin et al., 2017a; Lin et al., 2017b). Chidamide inhibits HDAC1, enhances histone acetylation at the DUSP1 promoter, and suppresses Smad3 signaling (Wang et al., 2025). Sulforaphane, via HDAC2 inhibition, upregulates BMP-7 expression through H3K9/14 acetylation and mitigates diabetic nephrofibrosis via the BMP7/Smad pathway (Kong et al., 2021). The HDAC3 inhibitor RGFP966 promotes PPARγ acetylation, increases Klotho, and inhibits TGF-β/Smad signaling (Chen et al., 2021; Lin et al., 2017b). HDAC4 inhibitors such as MC1568 and tacedinaline elevate histone H3 acetylation, suppress TGF-β1/Smad3 signaling, and restore Klotho expression (Shen et al., 2022; Xiong et al., 2019). Piceatannol downregulates HDAC4/5, inhibits the p38-MAPK pathway, and reduces ECM deposition (Choi et al., 2016). Selective HDAC6 inhibitors (ACY-1215, CKD-506, tubastatin A, and tubacin) enhance α-tubulin acetylation and inhibit NF-κB and TGF-β1/Smad3 pathways (Chen X. et al., 2020; Choi et al., 2018; Choi et al., 2015; Cebotaru et al., 2016). PCI34051, an HDAC8 inhibitor, restores cortactin acetylation and blocks TGF-β1/Smad3 signaling (Zhang Y. et al., 2020). Quisinostat, targeting HDAC11, restores Kruppel-like factor 15 (KLF15) activity and inhibits EMT (Mao et al., 2020).

**TABLE 4:** Small molecules targeting acetylation in CKD and their target proteins.

Small molecule	Targets	Phase	Experiental models	References
trichostatin A	HDAC1, HDAC2, HDAC3	Phase 2	UUO-induced mouse model Adenine-induced mouse model	Marumo et al. (2010) Lin et al. (2017a) Lin et al. (2017b)
Chidamide	HDAC1	Marketed	UUO-induced mouse model	Wang et al. (2025)
Sulforaphane	HDAC2	Phase 2	STZ-induced mouse model HG/Pal-induced HK11 cells	Kong et al. (2021)
RGFP966	HDAC3	Preclinical study	UUO-induced mouse model Adenine-induced mouse model	Chen et al. (2021) Lin et al. (2017b)
MC1568	HDAC4	Preclinical study	UUO-induced mouse model	Xiong et al. (2019)
Tasquinimod	HDAC4	Phase 3	UUO-induced mouse model	Shen et al. (2022)
Piceatannol	HDAC4, HDAC5	Phase 1	UUO-induced mouse model	Choi et al. (2016)
Tubacin	HDAC6	Preclinical study	ADPKD mouse model	Cebotaru et al. (2016)
Tubastatin A	HDAC6	Preclinical study	Angiotensin (ANG)-induced mouse model	Choi et al. (2015)
Rocilinostat (ACY-1215)	HDAC6	Preclinical study	UUO-induced mouse model	Chen et al. (2020b)
CKD-506	HDAC6	Phase 1	Systemic Lupus Erythematosus mouse model	Choi et al. (2018)
PCI34051	HDAC8	Preclinical study	UUO-induced mouse model	Zhang et al. (2020b)
Quisinostat	HDAC11	Early Phase 1	UUO)-induced mouse model HFD-induced mouse model Ang II- induced mouse model	Mao et al. (2020)
Honokiol	SIRT3	Phase 3	UUO-induced mouse model	Zhang et al. (2021b)
L002	p300	Pilot study	Ang II-induced hypertensive cardio-renal fibrosis mouse model	Rai et al. (2017)
C646	p300/CBP	Preclinical study	STZ-induced mouse model	Lazar et al. (2021)
Garcinol	PCAF	Phase 3	UUO-induced mouse model	Chung et al. (2019)

The Phase data for small molecules are derived from the Chinese Clinical Trial Registry (https://www.chictr.org.cn/index.html), the Drug Clinical Trial Registration and Information Disclosure Platform (http://www.chinadrugtrials.org.cn), ClinicalTrials.gov (https://clinicaltrials.gov), the ICTRP Search Portal (https://trialsearch.who.int/), and the official website of the National Medical Products Administration (https://www.nmpa.gov.cn).

Agents targeting HATs have also shown efficacy. Honokiol activates SIRT3, reduces mitochondrial protein acetylation, and improves energy metabolism (Zhang et al., 2021b). L002 and C646 competitively inhibit p300/CBP, reduce acetylation at H3K9, H4, and H3K27, and suppress the TGF-β/Smad pathway and oxidative stress (Rai et al., 2017; Lazar et al., 2021). Gambogic acid inhibits PCAF, reduces H3K9 acetylation, and suppresses NF-κB–mediated inflammation (Chung et al., 2019). It is encouraging that some ePTM-targeted compounds have entered the clinical stage. For instance, the HDAC inhibitor Chidamide, which is approved for peripheral T-cell lymphoma, has been marketed (Shi et al., 2017). Trichostatin A (Phase 3, for metastatic castration-resistant prostate cancer) and ACY-1215 (Phase 1/2, for lymphoma) are among those in clinical research stages (Sternberg et al., 2016; Amengual et al., 2021). However, that the approved indications for these drugs are mostly tumors or autoimmune diseases, and clinical trials specifically targeting CKD remain very limited. It should be clarified that acetylation imbalance is recognized as a driver of CKD progression, and targeted inhibition of relevant enzymes has demonstrated efficacy in UUO, DN, and adenine-induced pathological models. Importantly, some of these drugs, such as chidamide, undergo renal excretion, which underscores the necessity of further addressing safety concerns prior to their clinical translation for CKD.

## 4 Ubiquitination

### 4.1 Ubiquitination modification and its key enzymes involved

**FIGURE 3:**
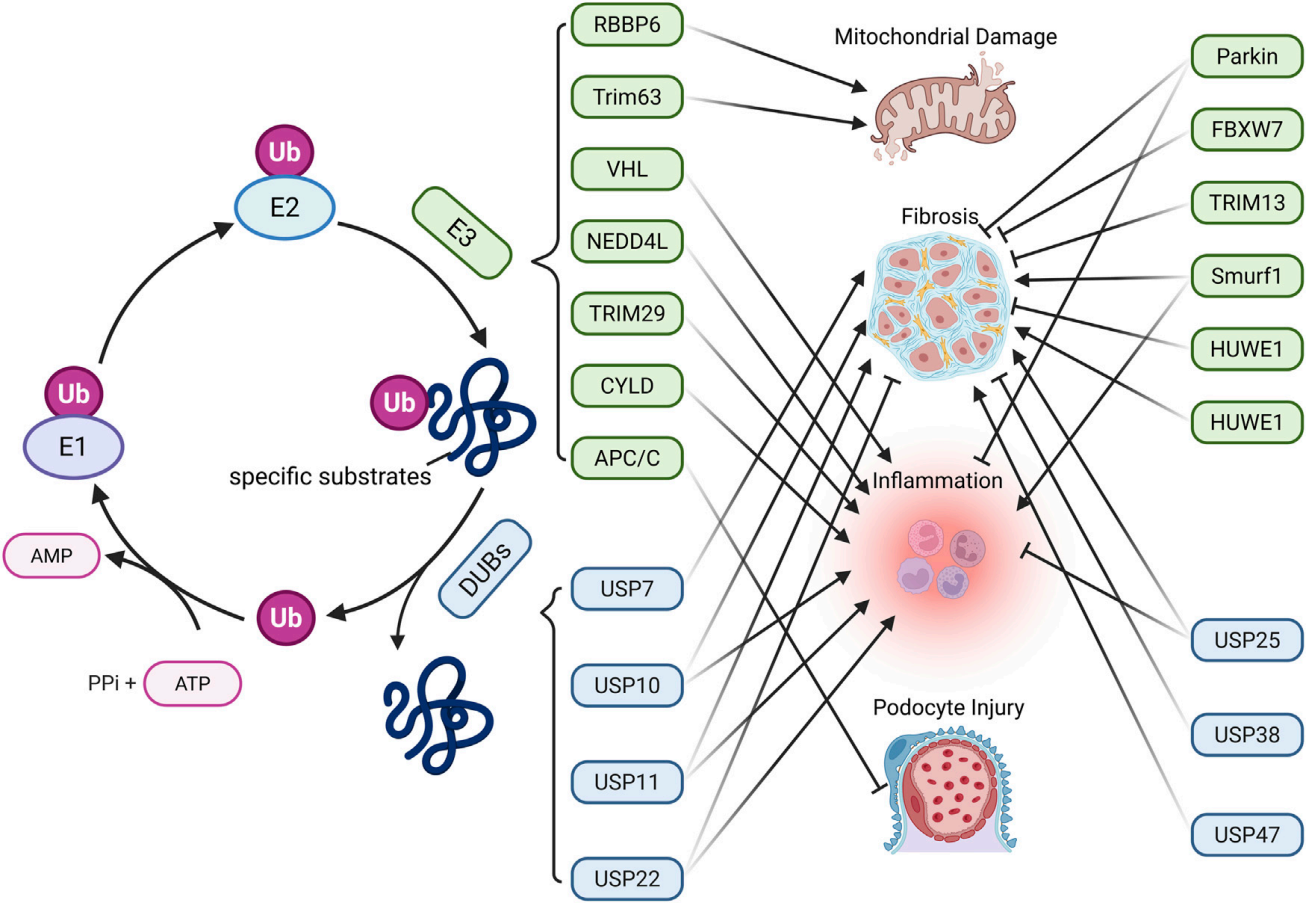
Main Mechanisms of ubiquitination-deubiquitination in CKD. Protein ubiquitination is mediated by ubiquitin-activating enzymes (E1), conjugating enzymes (E2), and ligases (E3). E1 activates Ub by forming a high-energy thioester bond with Ub in an ATP-dependent manner. The activated Ub is then transferred to E2 via a new thioester bond. Finally, E3 ligases facilitate the transfer of Ub to specific substrate proteins. Ubiquitinated proteins can be deubiquitinated by deubiquitinating enzymes (DUBs), enabling Ub recycling. The dynamic balance between protein ubiquitination and deubiquitination affects CKD progression through various pathways. (Created in https://BioRender.com).

Protein ubiquitination is a reversible process regulated by ubiquitinating enzymes (E1, E2, E3) and deubiquitinating enzymes (DUBs). First identified in 1975 (Nakamura, 2018), this modification is essential for maintaining renal cell function and homeostasis (Meyer-Schwesinger, 2019; You et al., 2024) E1 enzymes activate ubiquitin (Ub) in an ATP-dependent manner, forming a thioester bond. Ub is then transferred to E2 conjugating enzymes, and finally attached to target proteins by E3 ligases, which confer substrate specificity (Liao et al., 2024; Gan et al., 2025) (Figure 3). DUBs reverse this process by hydrolyzing Ub from proteins. Ubiquitination plays a central role in protein degradation via the ubiquitin–proteasome system, where Ub-tagged proteins are recognized and degraded by the proteasome (Meyer-Schwesinger, 2019). In human cells, over 50 E2 enzymes and approximately 600 E3 ligases have been identified (Wang M. et al., 2020).

### 4.2 Effect of ubiquitination in the pathological progression of CKD

Ubiquitination regulates renal fibrosis, inflammation, and sodium homeostasis in CKD by modulating key signaling pathways and cellular functions (Table 5). Among ubiquitination-related enzymes, E3 ligases are the most extensively studied. In DN, E3 ligases such as retinoblastoma binding protein 6 (RBBP6) (Hu et al., 2024), Von Hippel-Lindau (VHL) (Wang et al., 2019), tripartite motif containing (TRIM) 63 (Chen et al., 2023), NEDD4-like E3 ubiquitin protein ligase (NEDD4L) (Zhang et al., 2024), TRIM29 (Xu et al., 2023), SMAD-specific E3 ubiquitin protein ligase (SMURF) 1 (Lin et al., 2023), anaphase-promoting complex/cyclosome (APC/C) (Su et al., 2015), TRIM13 (Li et al., 2020), parkinson juvenile disease protein 2 (Parkin) (Chen K. et al., 2020), and F-Box and WD Repeat Domain Containing 7 (FBXW7) (Li et al., 2021) mediate the ubiquitination of targets including estrogen-related receptor alpha (ERRα), glucose-6-phosphate dehydrogenase (G6PD), PPARα, IkappaB kinase (IKK), IκBα, Takeda G protein-coupled receptor 5 (TGR5), nuclear factor erythroid 2-related factor 2 (Nrf2), C/EBP homologous protein (CHOP), cyclin B1, S-phase kinase-associated protein 2 (Skp2), Kelch-like ECH associated protein 1 (Keap1), GATA-binding protein 4 (GATA4), and SREBP-1 through distinct domains. These ubiquitination events contribute to mitochondrial dysfunction, oxidative stress, inflammation, and fibrosis.

**TABLE 5:** Regulatory networks and pathological effects of ubiquitination in CKD.

Involved Enzymes	Involved Proteins and modification change	Pharmacological effects	Involved pathways	Pathological model	References
RBBP6	ERRα ↑	RBBP6 promotes K48-linked ubiquitination and degradation of ERRα at the K100 site, downregulates mitochondrial-related proteins such as PGC1α, TOM20, and OXPHOS components, leading to mitochondrial structural damage and dysfunction	mitochondrial respiratory chain	Db/db mouse model STZ-induced mouse model	Hu et al. (2024)
VHL	G6PD ↑	Upon ubiquitination and degradation of G6PD, the production of NADPH is decreased, leading to the weakened function of antioxidant systems (such as GSH/GSSG) and the accumulation of ROS.	Glutathione metabolism pathway	Diabetic patients STZ-induced rat model STZ-induced mouse model G6PD-deficient mouse model	Wang et al. (2019)
Trim63	PPARα ↑	Upon ubiquitination and degradation of PPARα, the expression of its target proteins such as CPT1A and ACOX1 is reduced, leading to impaired fatty acid oxidation, lipid accumulation, and mitochondrial dysfunction (e.g., decreased expression of PGC-1α and COX1)	Fatty acid degradation pathway PPAR pathway Peroxisome pathway	Adrenomedullin-induced Nephropathy model Db/db mice model 5/6 Nx model	Chen et al. (2023)
NEDD4L	IKK ↑	NEDD4L mediates the ubiquitination and degradation of IκB Kinase, activates the NF-κB signaling pathway, promotes the expression of pro-inflammatory factors such as IL-6 and TNF-α, and induces podocyte inflammatory injury	NF-κB pathway	HFD/STZ-inducedrat model HG -stimulated podocyte model	Zhang et al. (2024)
TRIM29	IκBα ↑	TRIM29 mediates the ubiquitination and degradation of IκBα, relieves its inhibition on NF-κB, promotes the nuclear translocation of NF-κB and activates the NLRP3 inflammasome, thereby promoting the generation of GSDMD-N, the activation of Caspase-1 and the release of IL-1β/IL-18, and finally leading to podocyte pyroptosis	NF-κB pathway	HG -treated MPC5 podocyte model	Xu et al. (2023)
CYLD	IκBα ↑	Low CYLD expression increases the ubiquitination and degradation of IκBα, releases NF-κB to the nucleus, activates the transcription of MCP-1, IL-6, and IL-8	NF-κB pathway	HG -treated SV40 MES 13 cells model HG -treated HBZY-1 cells model	Li et al. (2017b)
APC/C	cyclin B1 ↓, Skp2 ↓	MAD2B inhibits the activity of APC/C, reduces the ubiquitination and degradation of cyclin B1 and Skp2, triggers cell injury and apoptosis	APC/C-Cdc20/Cdh1 pathway	Db/db mice model	Su et al. (2015)
Parkin	GATA4 ↑	Parkin mediates the ubiquitination and degradation of GATA4, inhibits GAS1 transcription, and thereby suppresses IL-6 and TGF-β1 and the deposition of ECM.	TGF-β1/Smad pathway	STZ-induced mouse model DN patients	Chen et al. (2020a)
FBXW7	SREBP-1 ↓	HG inhibits the expression of FBXW7 in renal tubular epithelial cells by activating the PI3K/Akt pathway, weakening its ubiquitination and degradation of SREBP-1, leading to the accumulation of SREBP-1 and activation of downstream proteins such as FASN and ACC, while inducing the deposition of ECM proteins	PI3K/Akt pathway	STZ-induced mouse model HG-induced HK cells	Li et al. (2021)
TRIM13	CHOP ↓	Low expression of TRIM13 weakens the ubiquitination and degradation of CHOP, promoting CHOP-mediated transcription of collagen synthesis-related genes such as Col1a2, Col4a1, and TGF-β1	TGF-β/Smad pathway	STZ-induced mouse model TGF-β1-stimulated cell model	Li et al. (2020)
SMURF1	TGR5 ↑	SMURF1 induces ubiquitination of TGR5 at the K306 site and promotes its degradation, thereby enhancing the expression of fibrogenic and inflammatory factors such as α-SMA and ICAM-1	TGF-β/Smad pathway NF-κB pathway	STZ/HFD-induced mouse model HG-treated Glomerular Mesangial Cells model	Lin et al. (2023)
	Nrf2 ↑, Keap1 ↑	Smurf1 promotes the ubiquitination of Nrf2 and Keap1, inhibits the Nrf2/ARE pathway, and induces the overexpression of FN and ICAM-1	Nrf2/ARE pathway	STZ-induced mouse model HG-treated GMC model	Gong et al. (2018)
HUWE1	EGFR ↓	The decrease in HUWE1 expression weakens its ability to promote EGFR ubiquitination and degradation, and the sustained activation of EGFR initiates downstream TGF-β1/Smad3, STAT3, and ERK1/2 signaling pathways, inducing the overexpression of ECM.	TGF-β1/Smad pathway JAK/STAT pathway ERK1/2 pathway	UUO-induced model	Zhu et al. (2020a)
TRAF6	KLF5 ↑	TRAF6 mediates K63-linked ubiquitination of KLF5 at K99 and K100 sites, promotes TIMP3 gene transcription, inhibits MMP activity, and reduces degradation of ECM (such as FN and COL-I)	ECM-receptor interaction pathway TNF signaling pathway	Rat Thy-1 nephritis model Mesangial Proliferative Glomerulonephritis model	Ying et al. (2023)
USP7	RUNX2 ↑	USP7 promotes the expression of osteogenic markers ALP, collagen 1 and osteocalcin through deubiquitination of RUNX2	Wnt/β-catenin pathway BMP pathway	5/6 Nx CKD-mineral and bone disorder mouse model	Lan et al. (2025)
USP10	P53 ↓	USP10 promotes the deubiquitination of P53, activates P21, and then upregulates fibronectin and α-SMA, while enhancing the release of inflammatory factors such as IL-1β and IL-6	TGF-β/Smad pathway NF-κB pathway	UUO-induced model TGF-β1-induced cell model	Liu et al. (2025b)
USP11	EGFR ↑	USP11 inhibits the deubiquitination of EGFR, activates the TGF-β1/Smad3, STAT3, and ERK1/2 pathways, induces the expression of α-SMA, collagen I, TGF-β1, CTGF, IL-1β and IL-18	TGF-β1/Smad3 STAT3 pathway ERK1/2 pathway	HN model FA-induced kidney fibrosis model	Shi et al. (2023)
	Tgfbr2 ↑	USP11 inhibits Tgfbr2 deubiquitination modification, activates Smad3 phosphorylation and the expression of P53 and P21, while downregulating Klotho, and promotes the production of fibronectin and α-SMA.	TGF-β1/Smad3 pathway	UUO-induced model FA-induced model	Ni et al. (2023)
	KLF4 ↓	USP11 inhibits KLF4 Ub degradation, activates the Caspase-1/GSDMD-N and Caspase-3/GSDME-N pathways, induces pyroptosis of renal tubular epithelial cells, and promotes the release of inflammatory factors IL-1β and IL-18 as well as the deposition of ECM components fibronectin and α-SMA.	Caspase-1/GSDMD-N pathway	UUO-induced model Ang II-induced Mouse Tracheal Epithlial Cells model	Wang et al. (2024a)
USP22	Sirt1 ↑	Reduced USP22 expression leads to K48-linked ubiquitination and degradation of Sirt1, resulting in transcriptional activation of fibrogenic factors such as FN and TGF-β1	TGF-β1/Smad pathway	AGEs-treated GMC model STZ-induced rat model	Huang et al. (2015b)
	HMGB1 ↓	USP22 promotes the deubiquitination of HMGB1, activates the NF-κB pathway, upregulates inflammatory factors such as TNF-α, IL-6, and IL-1β, and simultaneously promotes podocyte apoptosis and the expression of α-SMA and fibronectin in renal proximal tubular epithelial cells	NF-κB pathway	Ang II-induced mouse model Ang II-treated cell model	Peng et al. (2025)
USP25	TRAF6 ↑	USP25 alleviates renal fibrosis by deubiquitinating TRAF6, inhibiting TRAF6-mediated activation of NF-κB and MAPK pathways, and reducing the expression of proinflammatory factors TNF-α, IL-6 and fibrotic proteins fibronectin, collagen IV.	NF-κB pathway MAPK pathway	STZ-induced mouse model AGEs-treated SV40 MES-13 model AGEs-treated Bone Marrow-Derived Macrophages model	Liu et al. (2023a)
USP38	STRAP ↓	USP38 modulates the deubiquitination of STRAP, enhances the binding of STRAP to TGF-β receptors, promotes the phosphorylation of Smad2/3, and upregulates fibrotic proteins such as collagen I/III and α-SM.	TGF-β/Smad pathway	5/6 Nx model	Meng et al. (2025)
USP47	BTRC ↓, AKT1 ↓	USP47 inhibits the degradation of BTRC and AKT1 through deubiquitination, activates the AKT1 pathway, upregulates RUNX2, FGF23, and MGP, downregulates SM22α, and promotes VC.	AKT1 pathway	CKD-induced VC model	Xiao et al. (2022)

The target protein modification level change refers to the comparison between normal physiological conditions and CKD, pathological state. ↑ indicates an increase in the level of epigenetic modifications. ↓ indicates a decrease in the level of epigenetic modifications.

In CKD, DUBs such as cylindromatosis (CYLD), ubiquitin-specific protease (USP) 11, and USP22 are downregulated. CYLD and USP22 deubiquitinate IκBα, EGFR, and Sirt1, leading to NF-κB activation and partial EMT, thereby promoting fibrosis (Li Y. et al., 2017; Huang K. P. et al., 2015). In contrast, USP25 is upregulated as a compensatory mechanism and inhibits NF-κB and MAPK pathways by deubiquitinating TNF receptor associated factor 6 (TRAF6), delaying disease progression (Liu B. et al., 2023). In the UUO model, USP11 promotes renal fibrosis and tubular cell senescence by stabilizing TGFbeta type II receptor (Tgfbr2) and Kruppel-like factor 4 (KLF4) via deubiquitination, through Smad3/p53 signaling (Ni et al., 2023). It also contributes to pyroptosis through caspase activation (Wang X. et al., 2024). USP10 stabilizes p53, inducing tubular senescence and ECM accumulation (Liu S. et al., 2025). Conversely, Hect, uba, and wwe domain containing 1 (HUWE1) enhances EGFR degradation through ubiquitination, exerting anti-fibrotic effects (Zhu Q. et al., 2020). In hyperuricemic nephropathy (HN), USP11 and USP22 deubiquitinate EGFR and high mobility group box 1 (HMGB1), respectively. This modification activates inflammatory signaling and the TGF-β1/Smad3 pathway, thereby promoting renal fibrosis (Shi et al., 2023; Peng et al., 2025). In mesangial proliferative glomerulonephritis and the rat Thy-1 nephritis model, TRAF6 facilitates ECM accumulation by mediating K63-linked ubiquitination of KLF5 (Ying et al., 2023). In CKD-associated vascular calcification, USP47 stabilizes BTRC and AKT1 through deubiquitination, promoting osteogenic transdifferentiation of vascular smooth muscle cells (Xiao et al., 2022). In CKD-related atrial fibrillation, USP38 targets serine-threonine kinase receptor associated protein (STRAP) for deubiquitination, thereby activating the TGF-β/Smad pathway and contributing to atrial fibrosis (Meng et al., 2025). Conversely, in CKD-related mineral and bone disorders, USP7 deubiquitinates runt-related transcription factor 2 (RUNX2) and helps improve abnormal bone metabolism (Lan et al., 2025). Angiotensin II type 1 receptor (AT_1_R) is a key effector in the local renal renin-angiotensin system (RAS) (Zhu et al., 2019). In the hypertensive kidney injury model, tissue transglutaminase (TG2) modifies AT_1_R through isopeptide bonds, causing its accumulation and heightened sensitivity to angiotensin II, thus contributing to the model’s pathological processes, whereas the TG2 inhibitor ERW1041E can ease related symptoms (Liu C. et al., 2019). The dopamine 5 receptor (D_5_R) promotes degradation of glycosylated AT_1_R via the ubiquitin-proteasome pathway. D_5_R deficiency leads to increased AT_1_R expression and higher blood pressure, and the AT_1_R antagonist losartan can reverse this hypertension (Li et al., 2008). These findings underscore the central role of ubiquitination and deubiquitination in CKD progression and highlight potential therapeutic targets.

### 4.3 The therapeutic potential of ubiquitination in CKD

The development of E1 and E2 inhibitors remains limited due to their broad impact on numerous proteins and cellular networks throughout the body. Although small-molecule drugs targeting E3 ligases or DUBs have demonstrated therapeutic potential in both preclinical and clinical studies (Table 6), only a few have progressed to clinical trials, largely due to the complexity of target structures and the presence of multiple active sites.

**TABLE 6:** Small molecules targeting ubiquitination in CKD.

Small molecule	Targets	Phase*	Experiental models	References
Emodin	SYVN1	exploratory trials	CKD-induced VC model High-phosphate induced VSMCs model	Chen et al. (2025)
Vitamin K3	SIAH2	Marketed	LN model mice	Cheng et al. (2024)
Spautin-1	USP10	Preclinical study	UUO model	Liu et al. (2025b)
Mitoxantrone	USP11	Marketed	HN model FA-induced kidney fibrosis model UUO model	Shi et al. (2022), Ni et al. (2023), Wang et al. (2024a)
Quercetin	USP22/Snail1	Phase 1	Db/db mice HG-induced NRK-52E cells	Zhao et al. (2024a)

The Phase data for small molecules are derived from the Chinese Clinical Trial Registry (https://www.chictr.org.cn/index.html), the Drug Clinical Trial Registration and Information Disclosure Platform (http://www.chinadrugtrials.org.cn), ClinicalTrials.gov (https://clinicaltrials.gov), the ICTRP Search Portal (https://trialsearch.who.int/), and the official website of the National Medical Products Administration (https://www.nmpa.gov.cn).

Several DUB inhibitors have demonstrated renoprotective effects. Mitoxantrone, a USP11 inhibitor, prevents the deubiquitination of EGFR, Tgfbr2, and KLF4, thereby suppressing TGF-β1/Smad3, p53, and caspase-3/GSDME pathways. This reduces EMT, pyroptosis, and renal fibrosis (Shi et al., 2022; Ni et al., 2023; Wang X. et al., 2024). Quercetin inhibits USP22, promotes Snail1 degradation, and alleviates EMT and ECM accumulation in DN (Zhao X. et al., 2024). Spautin-1 enhances p53 ubiquitination by inhibiting USP10, suppressing pro-fibrotic and inflammatory gene expression in tubular epithelial cells (Liu S. et al., 2025). Targeting E3 ligases has also shown potential. Vitamin K3 inhibits seven in Absentia Homolog 2 (SIAH2), restores large tumor suppressor kinase 2 (LATS2) expression, and reduces Yes-associated protein (YAP) activity, thereby attenuating renal fibrosis in lupus nephritis (LN) (Cheng et al., 2024). Emodin enhances the interaction between the estrogen receptor and the E3 ligase SYVN1, promoting ERα ubiquitination and degradation, thereby alleviating vascular calcification in CKD (Chen et al., 2025). In addition, some ubiquitin-proteasome system inhibitors such as bortezomib and carfilzomib block the 26S proteasome, suppressing NF-κB and TGF-β/Smad signaling and reducing ECM accumulation, thus mitigating renal fibrosis (Sawa-Aihara et al., 2023; Zeniya et al., 2017). Although therapeutic strategies targeting ubiquitination still face challenges, the renoprotective effects of related inhibitors in pathological states such as DN, HN, and LN models, indicate that abnormal ubiquitination is a driver of CKD progression under these pathological conditions. Currently, Vitamin K3 is approved for the treatment of bleeding caused by vitamin K deficiency, and Mitoxantrone is approved for the treatment of various tumors (Dezee et al., 2006; Deng et al., 2021). The elimination of both in the body involves the kidneys, and if used for the treatment of chronic kidney disease, potential safety risks may exist based on their pharmacokinetics (PK) (Ehninger et al., 1990; Hassan, 2013).

## 5 Glycosylation

### 5.1 Glycosylation modification and its key enzymes involved

Glycosylation plays diverse and complex roles in CKD pathogenesis. Intracellular glycosylation mainly includes four types: N-glycosylation (linked to asparagine), O-glycosylation (linked to serine or threonine), C-glycosylation (linked to tryptophan), and glycosylphosphatidylinositol (GPI)-anchored glycosylation, with N- and O-glycosylation being the most common forms (He M. et al., 2024).

**FIGURE 4:**
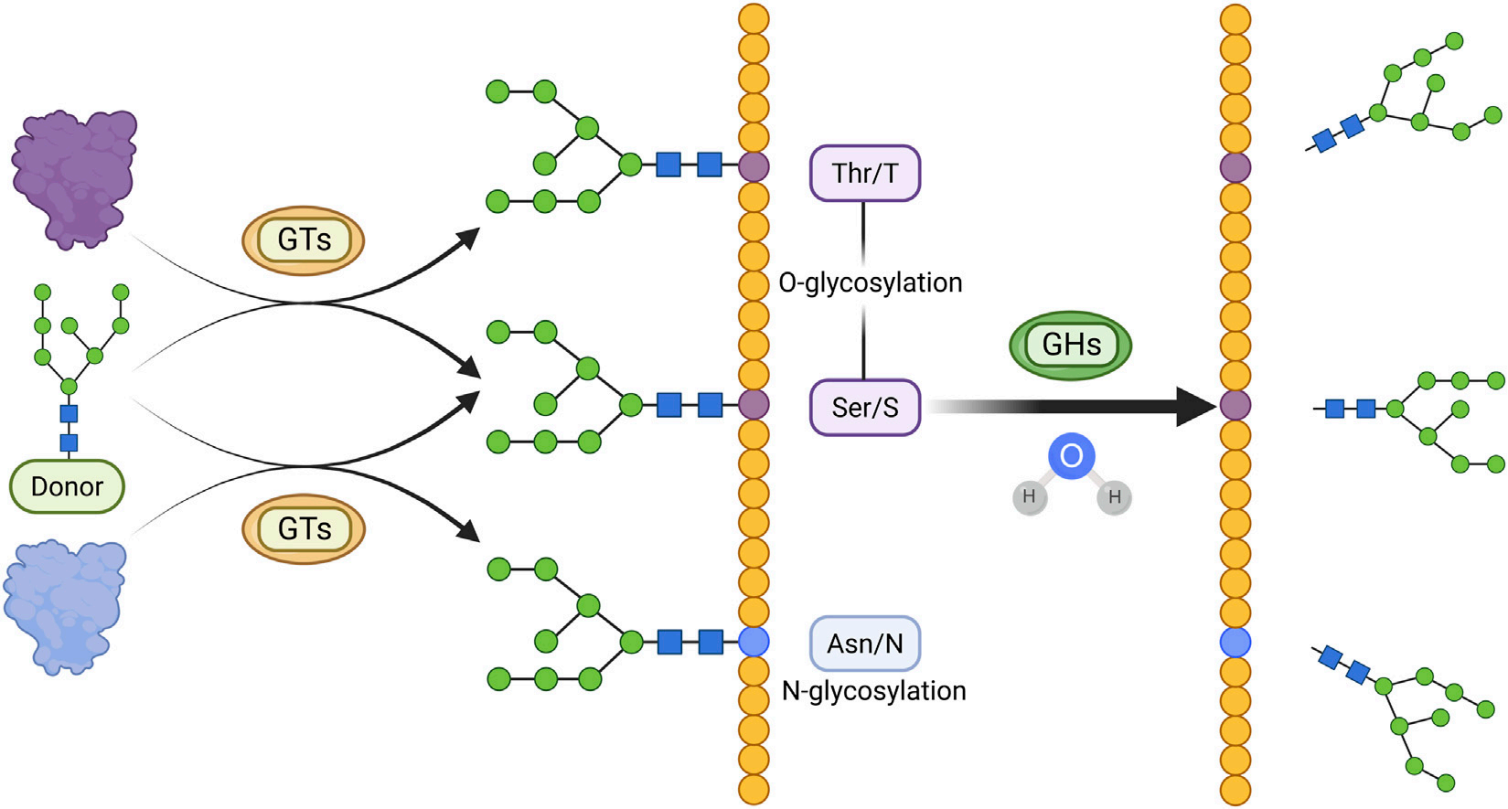
Main Mechanisms of glycosylation in CKD. Glycosyl groups are transferred from activated sugar donors to threonine/serine or asparagine residues by GTs, resulting in O-glycosylation or N-glycosylation, respectively. Glycosylated proteins can undergo hydrolysis of glycosidic bonds catalyzed by GHs. (Created in https://BioRender.com).

Two key enzyme classes regulate glycosylation in CKD: glycosyltransferases (GTs) and glycosidases (GHs) (Figure 4). GTs catalyze the transfer of sugar moieties from activated donors (e.g., UDP-Gal, GDP-Man) to acceptors such as proteins, lipids, and nucleic acids (Lairson et al., 2008). For example, α-1,6-fucosyltransferase mediates N-glycosylation of TGF-β receptor II in UUO models (Shen et al., 2013). Structurally, GTs are classified into GT-A, GT-B, and GT-C families (Lairson et al., 2008). GHs, or glycoside hydrolases, cleave glycosidic bonds by hydrolyzing the linkage between sugar residues or between sugars and aglycones (Cantarel et al., 2009). The GH family is large, comprising 191 subfamilies (GH1–GH191) and nearly two million annotated modules according to the CAZy database (https://www.cazy.org/Glycoside-Hydrolases.html). Alterations in GH activity are closely associated with disease progression. For example, in IgA nephropathy, elevated urinary levels of N-acetyl-β-D-glucosaminidase, released from injured proximal tubular epithelial cells, serve as a biomarker for tubulointerstitial involvement and renal function decline (Liu X. et al., 2023).

### 5.2 Effect of glycosylation in the pathological progression of CKD

Studies have revealed widespread alterations in the glycosylation profiles of plasma, urine, and kidney tissues in CKD, suggesting potential diagnostic utility. A clinical study identified 62 glycoproteins and 172 N-glycopeptides altered in individuals prior to clinical CKD onset (Santiago-Hernandez et al., 2024). Glycosylation of insulin-like growth factor Ⅱ correlates with age and eGFR, offering potential as a marker for disease progression and cardiorenal risk (Lohia et al., 2023). Specific lipid glycosylation patterns, such as decreased plasma lactosylceramide and elevated C18:1-hexosylceramide, are associated with macroalbuminuria and CKD progression, respectively (Lopes-Virella et al., 2024). A cross-sectional study identified three N-glycans (GP12, GP16, and GP22) as being associated with renal function (Adua et al., 2018). In patients with LN, abnormal cellular mannosylation has emerged as a potential biomarker capable of predicting CKD development (Alves et al., 2021). In IgA nephropathy (IgAN), reduced α-2,6-sialylation and increased galactose-deficient IgA1 (Gd-IgA1) are observed, though the prognostic value of Gd-IgA1 remains inconclusive (Ding et al., 2007; Vaz de Castro et al., 2024). Large-cohort analyses indicate that IgG glycosylation features, including altered galactosylation, sialylation, and bisecting GlcNAc, are associated with kidney function and improve disease prediction when combined with clinical data (Barrios et al., 2016; Zhao et al., 2024c). In animal models, both N- and O-glycan profiles of the renal brush-border membrane are altered in CKD and diabetes, with increased fucosylation and sialylation (Yu et al., 2021a; Yu et al., 2021b). These findings suggest that glycosylation patterns vary by disease subtype and may aid early detection and risk stratification.

Beyond biomarkers, glycosylation actively contributes to CKD pathogenesis, particularly in tubulointerstitial injury and fibrosis (Table 7). TGF-β1 enhances N-glycosylation of lysyl oxidase-like 2 (LOXL2) via N-acetylglucosaminyltransferase V (MGAT5), promoting fibrosis (Kamiya et al., 2023). Aberrant IgG glycosylation reduces Fcγ receptor binding and alters complement activation, contributing to inflammation in IgAN and membranous nephropathy (Barrios et al., 2016; Zhao et al., 2024c; Zhang et al., 2021c). In autosomal dominant polycystic kidney disease, mislocalization of ALG5 disrupts N-glycosylation and GPI anchoring of uromodulin (UMOD), leading to ER retention (Elhassan et al., 2024). In ESKD, peritoneal dialysis modifies transferrin (Tf) glycosylation, potentially impairing iron metabolism (Miljuš et al., 2024). Polypeptide N-acetylgalactosaminyltransferase 11 (Galnt11) deficiency reduces O-glycosylation of megalin, impairing proximal tubular reabsorption and causing proteinuria (Tian et al., 2019). Similarly, the hypertensive state can induce O-GlcNAcylation of megalin, reduce its surface expression, and inhibit the endocytosis and reabsorption of albumin and other proteins, ultimately leading to the development of proteinuria (Silva-Aguiar et al., 2018). In C57BL/6J mice with heminephrectomy, indoxyl sulfate promotes fibroblast growth factor 23 (FGF23) glycosylation and cardiac hypertrophy via the AhR–FGF23–FGFR4 axis (Kishimoto et al., 2023; Ho and Bergwitz, 2021). Under high-phosphate conditions, N- and O-glycosylation of cytochrome p450 27B1 (CYP27B1) enhances RUNX2 expression, while O-Linked N-acetylglucosamine transferase (OGT)-mediated YAP glycosylation inhibits autophagy, accelerating disease progression under hyperphosphatemia with CKD (Yimamu et al., 2022; Xu et al., 2020). In the renal proximal tubule epithelial cells of spontaneously hypertensive rats (SHR), high concentrations of H_2_O_2_ promote the accumulation of glycosylated AT_1_R in lipid rafts, thereby enhancing its sensitivity to Ang II, however, NADPH-oxidase inhibitor apocynin can reverse this phenomenon (Pedrosa et al., 2008). Given that hypertension is often accompanied by renal injury, it is speculated that such glycosylation modification may be potentially associated with renal injury, but the specific mechanism remains to be further confirmed by studies.

**TABLE 7:** Regulatory networks and pathological effects of glycosylation in CKD.

Involved proteins	Type and modification change	Pharmacological effects	Involved pathways	Pathological model	References
IgG	N-glycosylation ↓	N-glycosylation of IgG (including galactosylation, sialylation, core fucosylation and bisecting GlcNAc levels) can affect processes such as complement activation, inflammatory response and antibody-dependent cellular cytotoxicity by regulating its binding capacity to Fcγ receptors	Fcγ receptor-mediated immune effector pathway	Plasma samples from CKD Patients	Barrios et al. (2016) Zhao et al. (2024c) Zhang et al. (2021c)
Tf	N-glycosylation ↓	In ESKD patients, peritoneal dialysis induces changes in the glycosylation of Tf (increased core fucosylation and β-1,4-GlcNAc modification, decreased α-mannose and galactose modification)	—	Blood samples from CKD Patients	Miljuš et al. (2024)
FGF23	O-glycosylation ↑	Indoxyl sulfate activates the AhR, induces the expression of GALNT3 and HIF1α, promotes the glycosylation of FGF23, and activates the FGF23-FGFR4 signaling in cardiomyocytes	FGF23-FGFR4 pathway	C57BL/6J mice with heminephrectomy	Ho and Bergwitz (2021) Kishimoto et al. (2023)
CYP27B1	N-glycosylation ↑ O-glycosylation ↑	The N-glycosylation and O-glycosylation modifications of CYP27B1 are enhanced under hight-phosphate conditions, promoting the expression of osteogenesis - related genes such as RUNX2 in VSMCs and inducing the osteoblastic transdifferentiation of VSMCs by activating ER stress-related pathways	PI3K/Akt pathway Wnt pathway TGF-β pathway	Klotho mutant mouse model	Yimamu et al. (2022)
UMOD	N-glycosylation * GPI-anchored * glycosylation*	In kidney tissue of CKD patients, the abnormal localization of ALG5 leads to the abnormal N-glycosylation and GPI-anchored modification of UMOD, causing its pathological accumulation in the ER.	GPI-anchor biosynthesis pathway	Patients with ALG5 p.R79W Variant	Elhassan et al. (2024)
LOXL2	N-glycosylation ↑	TGF-β1 mediates the N-glycosylation modification of LOXL2 through MGAT5, which promotes collagen cross-linking and EMT, thereby exacerbating renal fibrosis	TGF-β1/Smad pathway	TGF-β1-induced Hexokinase2 cells	Kamiya et al. (2023)
Megalin	O-glycosylation ↓	Deficiency of Galnt11 leads to a decrease in the O-glycosylation of megalin, which in turn triggers low-molecular-weight proteinuria and causes specific defects in the reabsorption of vitamin D-binding protein, α1-microglobulin and retinol-binding protein by the proximal tubules	O-glycan biosynthesis pathway Endocytosis pathway	Galnt11^−/−^ mouse model	Tian et al. (2019)
OGT	O-glycosylation ↑	In SHR model, the expression of OGT is increased, which promotes the O-GlcNAcylation of megalin, reduces its surface expression, and inhibits albumin endocytosis and protein reabsorption	O-GlcNAc biosynthesis pathway	SHR rat model LLC-PK1 cell model	Silva-Aguiar et al. (2018)
YAP	O-glycosylation ↑	Under high-phosphate conditions, overexpressed OGT mediates the O-glycosylation of YAP. The OGT/YAP pathway upregulates the expression of Runx2 and downregulates α-SMA, thereby inducing the osteoblastic transdifferentiation of VSMCs	OGT/YAP pathway	5/6 Nx with high-phosphate diet-induced CKD rat model High-phosphate-induced rat VSMC calcification model	Xu et al. (2020)

The target protein modification level change refers to the comparison between normal physiological conditions and CKD, pathological state. ↑ indicates an increase in the level of epigenetic modifications. ↓ indicates a decrease in the level of epigenetic modifications. * indicates that there is no direct statement about the changes in the level of epigenetic modifications. —indicates that no relevant signaling pathway is mentioned.

### 5.3 The therapeutic potential of glycosylation in CKD

Given the regulatory role of glycosylation in CKD, targeting this process has gained attention as a potential therapeutic strategy. Several compounds have shown renoprotective effects in preclinical or clinical studies. In HK-2 cells, the MGAT5 inhibitor glucosamine hydrochloride reduces LOXL2 secretion by inhibiting its N-glycosylation, thereby attenuating TGF-β1-induced fibrosis (Kamiya et al., 2023). In spontaneously hypertensive rats (SHR), 6-diazo-5-oxo-L-norleucine inhibits lutamine-fructose-6-phosphate amidotransferase activity, decreases O-GlcNAcylation in the renal cortex, restores megalin localization, and reduces proteinuria (Silva-Aguiar et al., 2018). Tunicamycin, an N-glycosylation inhibitor, blocks tissue factor glycosylation and improves coagulation abnormalities in CKD (Humphries et al., 2021). In IgA nephropathy, prednisone reduces aberrant O-glycosylation of IgA1, possibly by modulating enzymes such as C1GalT1 and ST6GalNAc-II, though the mechanism remains unclear (Kosztyu et al., 2018). These indicate that abnormal glycosylation is involved in the progression of CKD under these pathological states, and interventions targeting glycosyltransferases, glycosidases, etc., are potential research mechanisms for the treatment of CKD.

However, the specific mechanisms by which glycosylation contributes to CKD pathophysiology have not yet been fully elucidated. Metabolic disorders commonly associated with CKD, such as diabetes, can exacerbate protein dysfunction through glycation under high-glucose conditions (Hoffmann et al., 2016), further complicating disease progression. Therefore, the development of therapeutic agents targeting glycosylation requires a comprehensive understanding of the specific roles of glycosylation in CKD. This includes distinguishing between protective and deleterious glycosylation events and accurately identifying therapeutic targets.

## 6 Lactylation

### 6.1 Enzymatic mechanisms of lactylation

Lactylation is a lactate-derived PTM involving the enzymatic transfer of a lactyl group to lysine residues, forming ester bonds and resulting in lactylated proteins (Li S. et al., 2025). This modification influences gene expression, signal transduction, and cellular metabolism by altering protein charge, conformation, and interaction patterns (Hou et al., 2025). Lactylation is dynamic and reversible, regulated by specific lactyltransferases (writers) and delactylases (erasers) (Fan et al., 2023).

**FIGURE 5:**
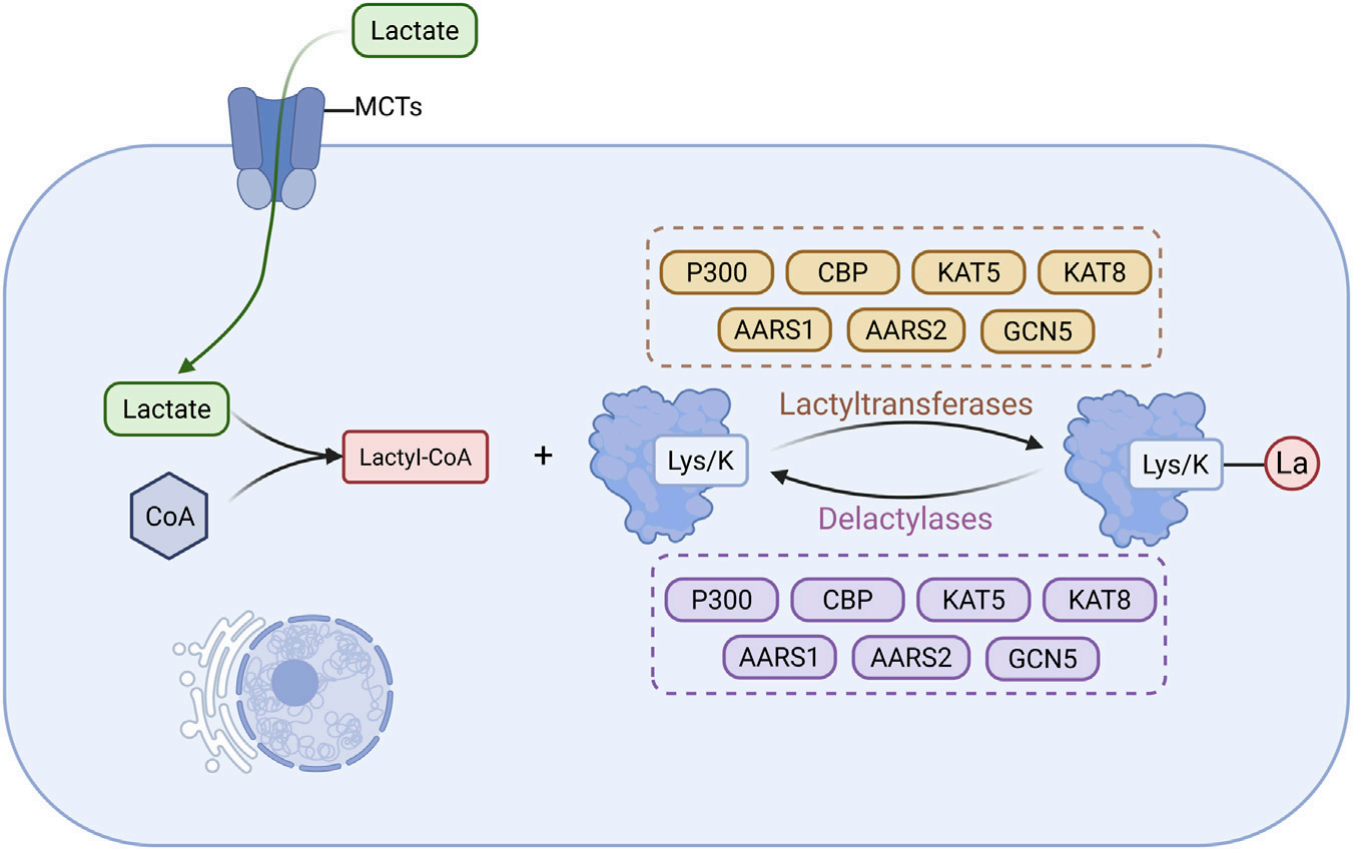
Main Mechanisms of lactylation in CKD. Lactate produced via glycolysis enters cells through monocarboxylate transporters to support the synthesis of lactyl-CoA, which subsequently serves as the donor of lactyl groups (La) that are enzymatically added to lysine residues on proteins by lactyltransferases. Lactylated proteins can also undergo delactylation through the action of delactylases. (Created in https://BioRender.com).

The donor molecule for lactylation is lactyl-CoA, synthesized by acyl-CoA synthetase, which catalyzes the conjugation of lactate with CoA (Li H. et al., 2024). Lactate, mainly produced via glycolysis, enters cells through monocarboxylate transporters (MCT1–MCT5), supporting intracellular lactyl-CoA production and linking cellular metabolism to epigenetic regulation (Wang Y. et al., 2024; Chen L. et al., 2022). Lactyltransferases catalyzing this modification include P300, CBP, lysine acetyltransferase (KAT) 5, KAT8, alanyl-tRNA synthetase (AARS) 1, AARS2, and GCN5, while delactylases such as HDAC1, HDAC2, HDAC3, HDAC8, and sirtuins (SIRT1, SIRT2, SIRT3) remove lactyl groups (Jin et al., 2023a; Iozzo et al., 2025; Zong et al., 2025). These enzymes modulate transcriptional programs and cellular functions by regulating the lactylation status of target proteins (Figure 5).

### 6.2 Effect of lactylation in the pathological progression of CKD

Research on protein lactylation in CKD is limited, with current studies mainly focusing on the impact of elevated lactate levels on fibrosis and inflammation (Li X. et al., 2025). Glycolytic enzymes contribute to CKD progression by promoting lactate accumulation and lactylation (Wang Y. et al., 2024). In FA nephropathy, phosphofructokinase-2/fructose-2,6-bisphosphatase 3 (PFKFB3) enhances glycolysis, increases lactate production, and induces H4K12 and H4K5 lactylation. This activates the NF-κB pathway and upregulates pro-inflammatory genes such as IκB, Rela, and Relb (Wang Y. et al., 2024). In the UUO model, pyruvate kinase M2 (PKM2) promotes H3K18 lactylation, enhances TGF-β1 transcription, and activates Smad3 signaling, facilitating macrophage-to-myofibroblast transition (Xiang et al., 2024). Meanwhile, the gut microbiota metabolite trimethylamine N-Oxide (TMAO) can alter the pyruvate metabolism of renal cells, leading to increased lactic acid accumulation, and then through the lactylation modification of histone H4, promote macrophage M2 polarization by directly binding to the promoters of IL-10 and TGF-β, resulting renal fibrosis (Tang et al., 2025). Whereas knockout of lactate transferase can reduce M2 macrophage infiltration and renal fibrosis (Tang et al., 2025). In diabetes, lactate induces K182 lactylation of ACSF2, reducing its activity and increasing mitochondrial reactive oxygen species (ROS) (Chen J. et al., 2024). In ischemic-reperfusion injury (IRI), citrate synthase lactylation impairs its function, activates the NLRP3 inflammasome, and accelerates AKI to CKD progression (Chen, 2022). These findings suggest that lactylation contributes to CKD by promoting inflammation, fibrosis, and mitochondrial dysfunction. Targeting glycolytic enzymes or interfering with lactylation may offer therapeutic potential.

### 6.3 The therapeutic potential of lactylation in CKD

In CKD models, several compounds have shown therapeutic potential by modulating lactate transport, glycolytic activity, or enzymes involved in protein lactylation (Nishima and Tanaka, 2024). Glycolysis inhibitors, such as oxamate (LDH inhibitor) (Ye et al., 2016), 3PO (Clem et al., 2008) and PFK15 (Clem et al., 2013) (PFKFB3 inhibitors), and shikonin (PKM2 inhibitor) (Xiang et al., 2025), suppress lactate production, leading to reduced histone lactylation (e.g., H3K18la and H4K12la), downregulates the NF-κB and TGF-β1/Smad3 signaling pathways and inhibits macrophage-to-myofibroblast transition (Wang Y. et al., 2024; Nishima and Tanaka, 2024). Inhibition of glucose transporter 1 (GLUT1) with BAY-876 lowers intracellular lactate levels and decreases H4K12la in renal tubular epithelial cells (Qiao et al., 2024). Additionally, HDAC inhibitors such as trichostatin A (TSA) and RGFP966 enhance delactylation and suppress NF-κB-mediated gene expression by reducing H4K12la levels (Cianciolo Cosentino et al., 2013; Chen et al., 2021). This indicates that lactylation modifications play a driving role in the occurrence and development of CKD and can serve as a new strategy for the treatment of chronic kidney disease.

## 7 Palmitoylation

**FIGURE 6:**
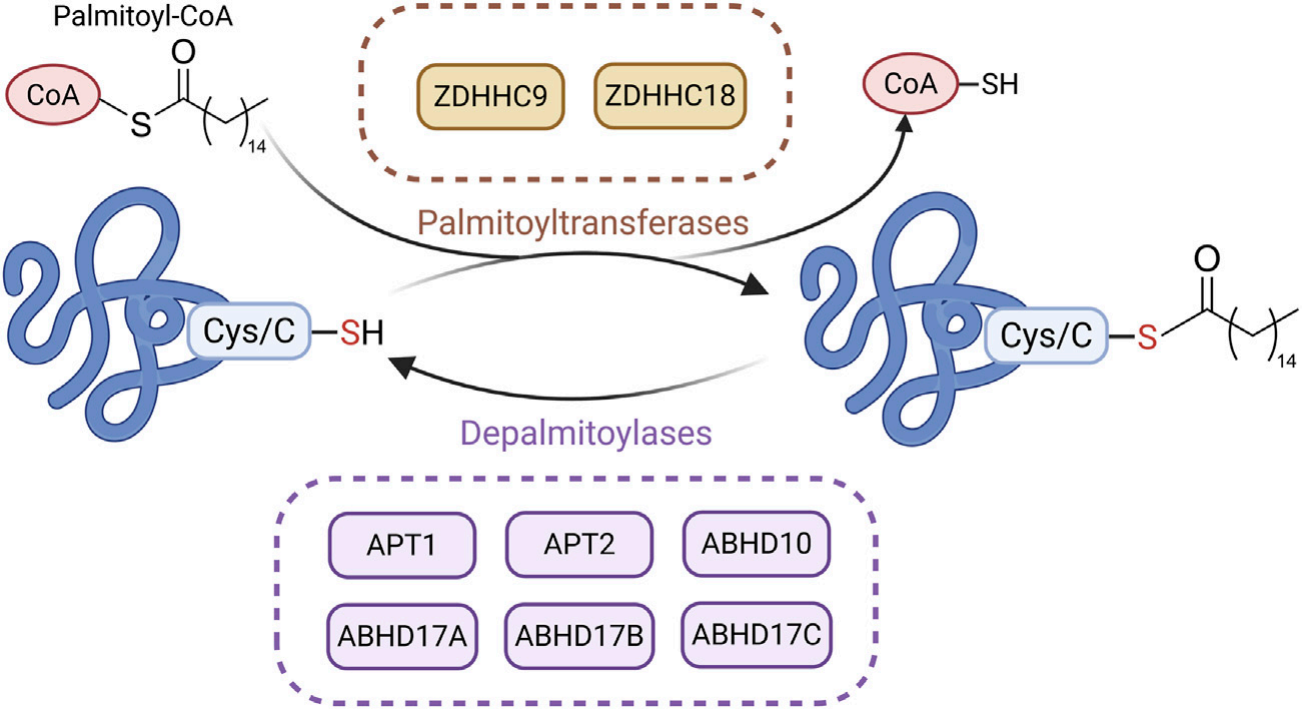
Main Mechanisms of palmitoylation in CKD. Under the action of palmitoyltransferases, palmitate is transferred from palmitoyl-CoA to the cysteine residues of substrate proteins via thioester bonds. Palmitoylated proteins can undergo depalmitoylation through the hydrolytic removal of palmitate by depalmitoylases. (Created in https://BioRender.com).

Palmitoylation is a reversible post-translational modification involving the covalent attachment of a 16-carbon palmitate to cysteine residues via thioester bonds. Although relatively underexplored in CKD, this modification increases protein hydrophobicity and affects localization, stability, and protein–protein interactions (Fraser et al., 2020; Fransisco et al., 2024). Palmitoylation is dynamically regulated by palmitoyltransferases (PATs) and depalmitoylases (Pei and Piao, 2024). Most PATs belong to the DHHC (Asp-His-His-Cys) family, with 23 DHHC enzymes identified in human cells, each showing distinct localization and substrate specificity (Tabaczar et al., 2017; De and Sadhukhan, 2018). T These enzymes transfer palmitate from palmitoyl-CoA to target proteins (Ko and Dixon, 2018). Depalmitoylases, such as acyl protein thioesterase (APT) 1, APT2, and α/β-hydrolase domain proteins (e.g., alpha/beta hydrolase fold domain (ABHD) 10, ABHD17A/B/C), remove palmitate by cleaving the thioester bond (Zhang and Hang, 2017; Cao et al., 2019) (Figure 6).

Recent studies suggest that palmitoylation plays a regulatory role in CKD progression. In UUO and IRI models, APT1 enhances β-catenin accumulation through depalmitoylation, thereby activating Wnt/β-catenin signaling and promoting fibrosis. This process can be blocked by the APT1 inhibitor ML348 (Gu M. et al., 2023). In contrast, DHHC9 promotes β-catenin palmitoylation, suppressing wingless-type MMTV integration site family (Wnt) signaling and reducing fibrosis (Gu M. et al., 2023). In UUO and FA-induced models, zinc finger DHHC-type containing (ZDHHC) 18 is upregulated and mediates Harvey rat sarcoma viral oncogene homolog (HRAS) palmitoylation, activating the MEK/ERK pathway and promoting EMT and fibrosis. Inhibiting ZDHHC18 or mutating HRAS palmitoylation sites alleviates fibrosis (Lu et al., 2025). Although research on palmitoylation in CKD is still in its early stages, these findings indicate that abnormal palmitoylation drives CKD progression in models such as UUO, IRI, and FA, thereby highlighting palmitoylation as a potential therapeutic target in CKD.

## 8 Crotonylation

**FIGURE 7:**
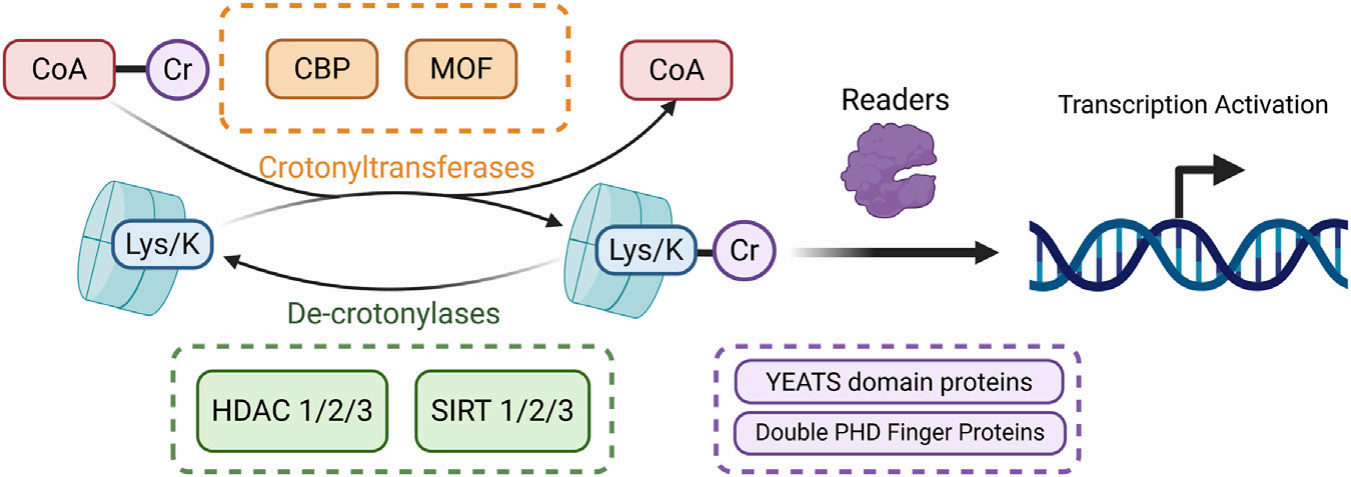
Mechanism of histone crotonylation. The crotonyl group is transferred from Cr-CoA to lysine residues on histones by crotonyltransferases, leading to histone crotonylation. The resulting crotonylated proteins are recognized by specific reader proteins (shown in purple), which facilitate transcriptional activation. Crotonylated proteins can be reverted through the removal of crotonyl groups by de-crotonylases, completing the dynamic regulation of this modification. (Created in https://BioRender.com).

Crotonylation is a histone lysine acylation modification first identified in 2011. It regulates gene expression by transferring a crotonoyl group (–CO–CH = CH–CH_3_) to lysine residues on histones, such as H3K9 and H3K18 (Tan et al., 2011). This process is dynamically controlled by a “writer–eraser–reader” system. Crotonyl-CoA (Cr-CoA) serves as the donor molecule, and histone crotonyltransferases, including p300/CBP and metal-organic framework (MOF), catalyze the modification. De-crotonylases such as HDAC1/2/3 and SIRT1/2/3 remove it, while reader proteins with the Yaf9, ENL, AF9, Taf14, Sas5 (YEATS) domains (e.g., ALL1-fused gene from chromosome 9 (AF9), YEATS2) or double plant homeodomain (PHD) fingers (e.g., monocytic leukemia zinc finger protein (MOZ), double PHD fingers 2 (DPF2) recognize crotonylated lysines and mediate transcriptional activation (Xie et al., 2024) (Figure 7).

Emerging evidence suggests that crotonylation contributes to CKD progression via multiple pathways. In patients with chronic kidney failure, 772 crotonylation sites are upregulated in peripheral blood mononuclear cells, with associated proteins enriched in pathways related to platelet granules and cell adhesion, implicating crotonylation in renal fibrosis (Huang et al., 2021). In a renal transplant ischemia–reperfusion model, neuropilin-1 (NRP1) is overexpressed and promotes energy metabolism dysfunction by reducing cytochrome Cox4i1 crotonylation via NF-κB activation. This aggravates fibrosis through the TGF-β/Smad3/PDGFβ pathway (Li Y. et al., 2024). In UUO and FA–induced models, acyl-CoA synthetase short-chain family member 2 (ACSS2, a Cr-CoA synthase) increases Cr-CoA production, enhances H3K9 crotonylation, and activates IL-1β transcription, leading to macrophage activation and tubular senescence. These effects can be reversed by the ACSS2 inhibitor VY-3-249, which attenuates fibrosis (Li L. et al., 2024). Conversely, in DN models, oral sodium crotonate increases H3K18 crotonylation via ACSS2, suppresses proinflammatory cytokines (IL-1β, IL-6), downregulates fibrotic markers (TGF-β1, α-SMA), and improves renal function (He Y. et al., 2024). Despite these findings, the overlap between crotonylation and other lysine modifications, such as acetylation, poses challenges for developing selective therapies. Moreover, the involvement of crotonylation in multiple interconnected pathways complicates the design of precise and safe interventions.

## 9 SUMOylation

**FIGURE 8:**
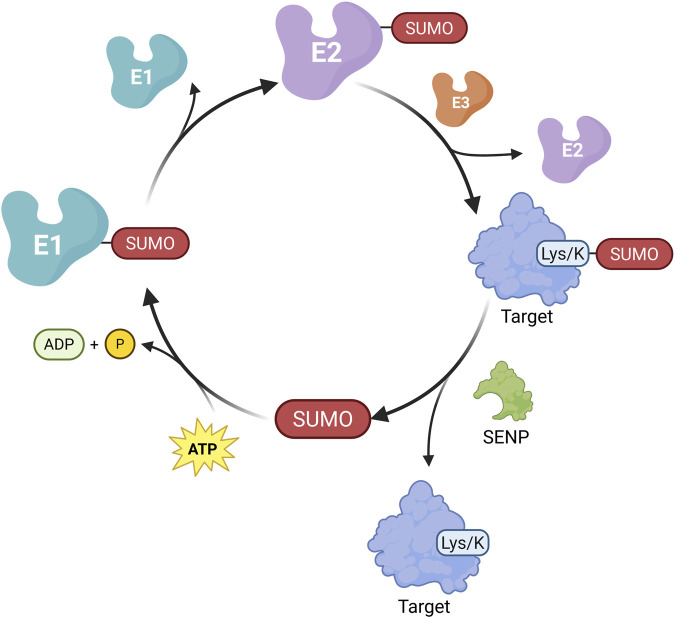
Mechanism of Protein SUMOylation. Similar to ubiquitination, the SUMOylation process is initiated by the activation of SUMO by the E1-activating enzyme in an ATP-dependent manner, forming a thioester bond between SUMO and E1. The activated SUMO is then transferred to the E2-conjugating enzyme. Subsequently, under the catalysis of an E3 ligase, SUMO is covalently attached to the lysine residue of the target protein, thereby completing the SUMOylation modification. SUMO-conjugated proteins can undergo deSUMOylation through the action of SENP, resulting in the removal of SUMO moieties. (Created in https://BioRender.com).

SUMOylation, similar to ubiquitination, is a post-translational modification in which the SUMO (Small Ubiquitin-like Modifier) protein is covalently attached to lysine residues of target proteins through a cascade involving E1, E2, and E3 enzymes (Sheng et al., 2021). DeSUMOylation and precursor processing are catalyzed by SUMO-specific protease (SENP, Figure 8) (Zhou H. et al., 2024). Five SUMO isoforms (SUMO1, SUMO2, SUMO3, SUMO4, and SUMO5) have been identified in mammalian cells, with most SUMOylated proteins located in the nucleus (Sheng et al., 2021; Sheng et al., 2019). In humans, the SENP family includes SENP1, SENP2, SENP3, SENP5, SENP6, and SENP7(Wu and Huang, 2023).

In CKD, SUMOylation is dysregulated and contributes to disease progression. In renal fibrosis models, such as UUO and unilateral ischemia-reperfusion injury, ATF4 binds to heat shock protein family A (Hsp70) member 5 (HSPA5) and promotes its SUMOylation, which exacerbates ferroptosis via the HSPA5 signaling pathway (Huang et al., 2025). β-catenin represses SUMO3 transcription, reducing SUMO3-mediated modification of live kinase B1 (LKB1), thereby impairing AMP-activated protein kinase (AMPK) activation and fatty acid oxidation (Chen S. et al., 2024). The E2 enzyme ubiquitin-conjugating enzyme 9 (UBC9) catalyzes the SUMOylation of the nuclear receptor nuclear receptor subfamily 5 group A member 2 (NR5A2), enhancing its binding to the calreticulin gene promoter, promoting its transcription, and upregulating the expression of fibrosis-related genes such as collagen type 1 alpha 1 (Col1α1) and TGFβ1 (Politis and Charonis, 2022; Arvaniti et al., 2016). In DN models, high glucose induces SUMOylation of IκBα, HIF-1α, Smad4, and STAT1, activating NF-κB and TGF-β signaling pathways and promoting inflammation and fibrosis (Huang et al., 2013; Wusiman et al., 2025; Gu C. et al., 2023; Zhou et al., 2014). High glucose also triggers the de-SUMOylation of RBMX, leading to mitochondrial dysfunction and contributing to tubulointerstitial fibrosis (Yang et al., 2024).

Targeting SUMOylation has shown potential in reducing renal fibrosis. For example, the UBC9 inhibitor 2-D08 specifically blocks NR5A2 SUMOylation, thereby attenuating fibrosis in the UUO model (Arvaniti et al., 2016). Some natural compounds, including Ginkgolic acid and Astragaloside IV, appear to influence SUMOylation, though their effects in CKD models remain unconfirmed (Wang B. S. et al., 2021; Yu et al., 2022).

## 10 Prenylation

**FIGURE 9:**
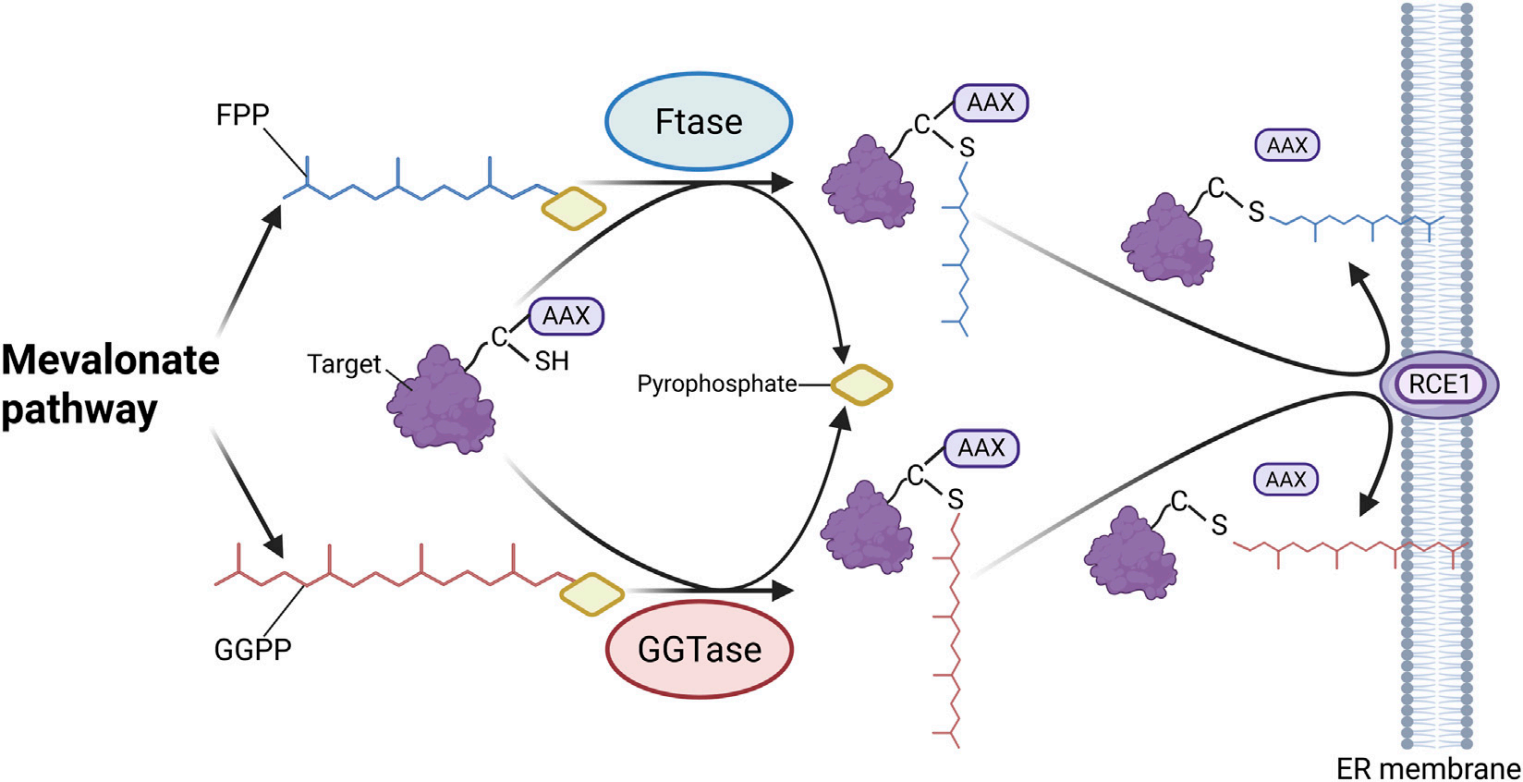
Mechanism of protein prenylation. Isoprenoid carriers, including FPP and GGPP, are synthesized via the mevalonate pathway. FTase and GGTase specifically recognize the CAAX (C represents cysteine) motif at the C-terminus of target proteins and catalyze the covalent attachment of farnesyl or geranylgeranyl groups to cysteine residues, anchoring the modified proteins to the endoplasmic reticulum membrane. Subsequently, the -AAX residues are cleaved by RAS-converting CAAX endopeptidase 1 (RCE1), completing the prenylation process. (Created in https://BioRender.com).

Recent studies have shown that protein prenylation is closely linked to CKD progression. This post-translational modification involves the covalent attachment of isoprenyl groups (farnesyl or geranylgeranyl) to the C-terminal CAAX (C represents cysteine, A refers to aliphatic amino acids, and X typically indicates methionine or serine) motif of target proteins (Mohamed et al., 2024; Khwaja et al., 2006) (Figure 9). Prenylation facilitates membrane anchoring and activates downstream signaling pathways (Khwaja et al., 2006). The process is mainly catalyzed by four types of prenyltransferases: farnesyltransferase (FTase), geranylgeranyltransferase (GGTase)-type I, GGTase type-II, and GGTase-III (Marchwicka et al., 2022) Their activity depends on intermediates produced by the mevalonate pathway, where HMG-CoA reductase acts as the rate-limiting enzyme (Khwaja et al., 2006; Buemi et al., 2002).

In fibrotic animal and cellular models, aberrant prenylation of proteins such as H-Ras, Ki-Ras, and RhoA has been shown to activate the NF-κB, TGF-β1/Smad, and MAPK signaling pathways, thereby promoting renal tubular EMT, fibroblast activation, and ECM deposition (Rodríguez-Peña et al., 2014; Sharpe et al., 1999; Khwaja et al., 2006). Small-molecule inhibitors targeting this process have shown therapeutic potential. Statins (e.g., lovastatin and atorvastatin) inhibit HMG-CoA reductase, reducing the synthesis of farnesyl pyrophosphate (FPP) and geranylgeranyl pyrophosphate (GGPP), and thereby suppressing Ras and RhoA prenylation. This attenuates mesangial cell proliferation, inflammation, and ECM accumulation (Essig et al., 1998; Buemi et al., 2002; Rodríguez-Peña et al., 2014). The FTase inhibitor L-744,832 directly blocks Ras farnesylation and reduces fibronectin levels (Rodríguez-Peña et al., 2014). GGTI-298, a GGTase inhibitor, disrupts RhoA-mediated Erk1/2 and Akt activation, thus limiting fibrosis (Khwaja et al., 2006). In the 5/6 Nx model, chaetomellic acid A selectively inhibits H-Ras farnesylation and alleviates glomerulosclerosis and arteriolosclerosis via the TGF-β1 pathway (Nogueira et al., 2017). Collectively, these findings support the potential of targeting dysregulated prenylation as an antifibrotic strategy in CKD.

## 11 Other ePTMs

In addition to the aforementioned ePTMs, other modifications, such as myristoylation, succinylation, and sulfenylation, also contribute to CKD progression by modulating protein function and interactions. Myristoylation involves the covalent attachment of myristic acid to the N-terminal glycine residue of a protein, a reaction catalyzed by N-myristoyltransferase (Wang B. et al., 2021). In both CKD patients and animal models, myristoylated alanine-rich C-kinase substrate shows altered expression and has been proposed as a potential biomarker for chronic renal injury, although its mechanistic role remains unclear (Kim et al., 2021).

Succinylation refers to the transfer of a succinyl group (–CO–CH_2_–CH_2_–CO–) to lysine residues, mediated by succinyltransferases such as SUCL2 and GCN5, while desuccinylation is regulated by enzymes like SIRT5 (Zhang et al., 2011). In mouse models of IRI, global lysine succinylation is elevated, whereas SIRT5 deficiency improves renal function, suggesting a role for succinylation in the transition from AKI to CKD (Chiba et al., 2019).

Sulfenylation is a PTM where cysteine thiols (-SH) are oxidized to sulfenic acids (-SOH), regulated by redox environments and related enzymes (Mu et al., 2024). Direct research on its association with CKD remains limited. In human kidney microsomes oxidative stress triggers sulfenylation of CYP2C8, CYP2D6, CYP3A4, and CYP4A11 (Albertolle et al., 2018; Albertolle et al., 2017). This may reduce their activity, impairing renal metabolism of endogenous substances and detoxification of exogenous drugs, thereby exacerbating kidney injury and contributing to the oxidative stress-metabolic disorder cycle in CKD. Under high glucose or diabetic conditions, sulfenylation at Cys358 of PKM2 inhibits its tetramer formation and activity which leads to accumulation of toxic glucose metabolites (e.g., methylglyoxal, sorbitol), mitochondrial dysfunction, and podocyte apoptosis, worsening renal pathology (Qi et al., 2017). Notably, the PKM2 activator TEPP-46 can reduce this modification, reversing damage (Qi et al., 2017). Thus, sulfenylation may participate in CKD progression by affecting renal metabolic enzymes and key signaling molecules, making its regulation a potential therapeutic target for CKD.

## 12 Crosstalk between different ePTMs

In CKD, ePTMs dynamically regulate protein function, signaling pathways, and gene expression, thereby contributing to renal fibrosis, inflammation, and metabolic dysregulation. Complex crosstalk occurs among distinct ePTMs, exhibiting model-specific regulatory patterns under different pathological contexts. Accordingly, elucidating the mechanisms of ePTM crosstalk across diverse CKD models is essential for a comprehensive understanding of disease progression and for the development of novel therapeutic strategies.

In DN, the interplay between methylation and other ePTMs appears to be particularly prominent. One mechanism involves the activities of methyltransferases are frequently regulated by ubiquitination and acetylation. For example, ubiquitination of histones H2AK119 and H2BK120 can increase the expression of the methyltransferases SET7/9 and SUV39H1, thereby enhancing H3K4 and H3K9 methylation, respectively, which promotes the transcription of fibrosis-related genes (Goru et al., 2016; Pan et al., 2024). In addition, the activity of the histone H3K4 methyltransferase Set1 is regulated by N-terminal acetylation mediated by N-acetyltransferases, which maintains H3K4 methylation, thereby activating the transcription of profibrotic genes and contributing to podocyte injury in DN (Woo et al., 2024; Sasaki et al., 2016; Zhang et al., 2023). Another mechanism arises from the competition between methylation and acetylation for the same target to influence disease progression. DOT1L, the only known H3K79 methyltransferase, competes with HDAC2 at the endothelin-1 promoter to regulate the balance between methylation and acetylation, thereby affecting fibrosis in DN (Zhang L. et al., 2020). Moreover, a novel ubiquitin-like modification, neddylation, has been shown to stabilize RhoA by preventing its ubiquitination, subsequently activating the ERK1/2 pathway and driving fibrosis (Li X. Q. et al., 2025).

In HN, reports on ePTM crosstalk are relatively limited and mainly involve phosphorylation, ubiquitination, and acetylation. For example, the deubiquitinase OTUD6A removes ubiquitin from STAT3, thereby enhancing its phosphorylation, nuclear translocation, and promotion of Ang II–induced fibrosis (Sun et al., 2024). In parallel, HDAC6 deacetylates Smad2/3, enhancing their phosphorylation and binding activity at profibrotic gene promoters, thus activating the TGF-β/Smad pathway (Choi et al., 2015). In HN, AT_1_R is a central regulatory molecule that mediates Ang II signaling to modulate renal hemodynamics, sodium reabsorption, and fibrosis, thereby influencing CKD progression (Wehbi et al., 2001). Its function is precisely regulated by glycosylation, ubiquitination, and phosphorylation. The phosphorylation state affects the efficiency and sites of glycosylation, while glycosylation may alter the receptor conformation to influence the exposure of phosphorylation sites, subsequently regulating the membrane localization and functional activity of AT_1_R, which affects disease progression under pathological conditions such as hypertension (Al-Qattan et al., 2006; Pedrosa et al., 2008). Additionally, phosphorylation can recruit E3 ubiquitin ligases to promote ubiquitination and degradation, while glycosylation may maintain receptor stability and affect the ubiquitination process (Liu C. et al., 2019; Li et al., 2008). Moreover, these three modifications precisely regulate the signal transduction specificity of AT_1_R through dynamic interactions. For instance, the phosphorylation “barcode” determines the downstream β-arrestin-mediated signaling pathway, glycosylation ensures the correct folding and localization of the receptor, and ubiquitination participates in receptor desensitization and degradation balance, collectively influencing its function under related pathological conditions (Gareri et al., 2024; Liu C. et al., 2019; Li et al., 2008).

In autoimmune kidney diseases, studies on ePTM crosstalk have so far been largely confined to LN. In LN, ePTM interactions play a critical role in regulating mesangial cell hyperproliferation and inflammatory responses. Recent study demonstrates that lactylation of PBX1 enhances its interaction with the E3 ubiquitin ligase TRIM21, thereby promoting PBX1 ubiquitination and degradation, which subsequently reduces the transcription of the cell cycle inhibitor P27 and drives abnormal mesangial cell proliferation (Liu E. et al., 2025). In addition, crotonylation competes with acetylation at the same lysine residues, thereby influencing inflammatory and fibrotic processes associated with autoimmune kidney disease (Zeng et al., 2023).

Renal fibrosis is a common pathological process across all types of CKD, and the UUO model, as a classic fibrosis model, has been widely used to investigate ePTM crosstalk in CKD. In the UUO model, multiple ePTMs orchestrate the fine-tuned regulation of fibrosis-related signaling pathways, particularly at the level of transcription factors and histone modifications. For instance, PRMT1 catalyzes the methylation of bromodomain protein 4 and enhances its phosphorylation, which in turn increases the acetylation of Snail, thereby promoting Snail-mediated EMT and fibrosis (Xiong C. et al., 2025). EZH2 aggravates fibrosis progression by downregulating PTEN and increasing STAT3 and ERK1/2 phosphorylation (Zhou et al., 2016). In contrast, JMJD3-mediated demethylation of H3K27me3 inhibits AKT and ERK1/2 phosphorylation, thereby suppressing fibrosis progression (Yu C. et al., 2021). At the signaling protein level, O-GlcNAcylation of serine/threonine kinase (RAF1) prevents its ubiquitination, stabilizing RAF1 and activating the Ras/RAF1/ERK pathway to drive fibrosis (Feng et al., 2020). Conversely, SIRT2 deacetylates SMAD2/3 and promotes their ubiquitination and degradation, exerting an antifibrotic effect (Yang et al., 2023). In contrast, HDACs promote fibrosis by DUSP1, which enhances Smad3 phosphorylation and activates the TGF-β/Smad pathway (Wang et al., 2025). Similarly, FAT10 overexpression increases checkpoint kinase 1 (CHK1) levels by reducing USP7 ubiquitination, thereby amplifying TGF-β signaling and promoting fibrosis (Shao et al., 2022). Palmitoylation also plays a critical role in the regulation of the TGF-β/Smad pathway. ZDHHC18-mediated palmitoylation of HRAS promotes downstream MEK/ERK phosphorylation and aggravates fibrosis (Lu et al., 2025), while downregulation of DHHC9 reduces palmitoylation and ubiquitination of β-catenin, leading to its accumulation and Smad2/3 activation, further exacerbating fibrosis (Gu M. et al., 2023). Moreover, macrophage polarization represents a decisive factor in UUO-induced fibrosis (Wehbi et al., 2001). Among macrophages, M1 macrophages secrete pro-inflammatory factors in the early stage, exacerbating renal injury, while M2 macrophages can promote tissue regeneration by secreting related factors, but their persistent infiltration can aggravate renal fibrosis (Gao et al., 2025; Tan et al., 2025). In this process, various epigenetic modifications, such as histone lactylation, acetylation, and methylation, regulate the expression of genes related to macrophage polarization, thereby influencing chronic kidney disease (Gao et al., 2025; Tang et al., 2025; An et al., 2023; Zhan et al., 2025). Meanwhile, there exist cross-regulations between metabolism and epigenetics (e.g., TMAO can induce lactate secretion and promote histone lactylation) and intercellular epigenetic signal transmission (e.g., regulation by exosomal non-coding RNAs). These regulations collectively participate in the pathological process of chronic kidney disease by modulating macrophage polarization (Tan et al., 2025; Tang et al., 2025; Zhan et al., 2025). Drugs targeting epigenetic regulation, such as SIRT6 agonists, JMJD3 inhibitors, and TMAO inhibitors, can regulate the balance of macrophage polarization, providing potential strategies for the treatment of CKD (Gao et al., 2025; Tang et al., 2025; An et al., 2023). In summary, ePTMs in the UUO model drive fibrosis not only through regulation of transcription factors and signaling pathways but also by shaping macrophage polarization, together forming a complex pathological network of renal fibrosis.

In summary, ePTM crosstalk in CKD forms a highly complex regulatory network, influencing disease progression through the modulation of protein expression and signal transduction. Elucidating these ePTM interactions not only deepens our understanding of the molecular drivers underlying CKD but also provides a theoretical foundation for the development of multi-target combination therapies.

## 13 Conclusion and future perspectives

EPTMs play a crucial role in the pathogenesis of CKD by regulating key pathological processes such as renal fibrosis, inflammation, metabolic dysregulation, and cellular stress responses (Laget et al., 2022). In this review, we summarize the mechanisms and clinical significance of major ePTMs, including methylation, acetylation, ubiquitination, enzymatic glycosylation, lactylation, palmitoylation, crotonylation, SUMOylation, and prenylation, in CKD. Despite increasing attention, most ePTMs in CKD remain in the early stages of investigation, and their complex regulatory networks and cell type-specific functions require further elucidation. The crosstalk between different ePTMs represents a critical yet underexplored aspect of CKD pathogenesis. This intricate interplay complicates therapeutic strategies, as targeting a single modification may inadvertently interfere with interconnected pathways and lead to unintended effects (Fontecha-Barriuso et al., 2018).

Emerging evidence highlights the therapeutic potential of targeting ePTMs in the treatment of CKD. Small-molecule inhibitors of methyltransferases (e.g., 3-DZNeP targeting EZH2 (Zhou et al., 2016)), HDACs (e.g., ACY-1215 targeting HDAC6 (Chen X. et al., 2020)), and ubiquitin-related enzymes (e.g., mitoxantrone targeting USP11 (Shi et al., 2023)) have shown encouraging effects in preclinical models by mitigating fibrosis, inflammation, and organ injury. In addition, agents targeting glycosylation (e.g., glucosamine hydrochloride (Kamiya et al., 2023)) and lactylation (e.g., shikonin (Xiang et al., 2025)) provide novel avenues for modulating CKD-associated pathways.

Despite these advances, several challenges hinder the clinical translation of ePTM-targeted therapies. First, the crosstalk among different ePTMs, including the overlapping enzymatic machinery of crotonylation and acetylation (Xie et al., 2024)) complicates selective targeting and increases the risk of off-target effects. Second, the regulatory networks of ePTMs are complex, with individual enzymes often acting on multiple substrates and pathways. For example, PRMT5 modulates both SREBP1 and NF-κB signaling (Zhang et al., 2018; Wei et al., 2013), highlighting the need for a more comprehensive understanding of context-specific functions. Third, most current findings are derived from animal models, and the safety and efficacy of ePTM-based interventions in humans remain to be rigorously validated. Notably, CKD patients with impaired renal function and metabolic disorders may face unique PK/pharmacodynamic (PK/PD) challenges, such as drug accumulation or altered target binding in fibrotic microenvironments, which further limit translational progress (Franchi et al., 2022). A notable example is the discontinuation of the JNK inhibitor CC-930 due to hepatotoxicity in clinical trials (Qian et al., 2023). Future research should aim to identify tissue- and cell-type-specific patterns of ePTMs to facilitate the development of precision medicine approaches. It is also essential to design highly selective inhibitors using advanced molecular technologies and to investigate combination therapies that target multiple ePTMs simultaneously, thereby enhancing therapeutic efficacy while minimizing adverse effects.

In conclusion, ePTMs represent a promising frontier in CKD research, bridging the gap between molecular mechanisms and clinical application. Among the discussed ePTMs, methylation, acetylation, and ubiquitination are relatively well-studied with more preclinical evidence supporting their therapeutic potential. With continued efforts to elucidate their biological roles and refine targeting strategies, ePTM-based therapies may offer substantial benefits for improving outcomes in patients with CKD and addressing current therapeutic limitations.
